# Atg39-dependent nucleophagy and Atg40-dependent ER-phagy require the Apq12/Brr6/Brl1 complex

**DOI:** 10.1073/pnas.2620353123

**Published:** 2026-09-30

**Authors:** Dongmei Liu, Shuliang Chen, Hua Yuan, Susan Ferro-Novick, Peter Novick

**Affiliations:** ^a^ Department of Cellular and Molecular Medicine, University of California San Diego, La Jolla, CA 92093-0668

**Keywords:** ER-phagy, nucleophagy, Apq12, Brr6, Brl1

## Abstract

Autophagic degradation of parts of the nucleus (nucleophagy) and endoplasmic reticulum (ER-phagy) utilizes the selective autophagy receptors, Atg39 and Atg40, that link their target structures to the autophagy machinery. Here, we show that a complex of three proteins, Apq12, Brr6, and Brl1, is needed for both Atg39-dependent nucleophagy and Atg40-dependent ER-phagy. Apq12, Brr6, and Brl1 each have two transmembrane domains flanking a luminal region that contains an amphipathic helix. The Apq12/Brr6/Brl1 complex has been implicated in membrane remodeling during nuclear pore formation. While autophagosomal flux is unaffected by the loss of Apq12, Brr6, or Brl1, vacuolar delivery of outer and inner nuclear membrane proteins (Hmg1, Nvj1, and Src1), pan-ER proteins (Sec63 and Per33), and an ER tubule junction protein (Lnp1) is blocked. In *apq12Δ* cells, Atg39 and Atg40 receptors concentrate in puncta and associate with the autophagic machinery; however, their delivery to the vacuole is blocked. Atg39 puncta extend into the cytoplasm but fail to detach from the nuclear envelope (NE), suggesting a defect in membrane scission. Coimmunoprecipitation and crosslinking analysis indicate that Atg39 and Atg40 interact with the Apq12/Brr6/Brl1 complex. We propose that the membrane remodeling activity of the Apq12/Brr6/Brl1 complex is needed to release fragments of the NE and ER so they can be sequestered within autophagosomes for degradation.

## Significance

Segments of the nucleus and endoplasmic reticulum (ER) are degraded by autophagy in response to starvation or the accumulation of misfolded proteins. These pathways are crucial for cellular homeostasis and defects lead to accelerated chronological aging. We demonstrate the requirement for a complex of three proteins, Apq12, Brr6, and Brl1 on both pathways. Our analysis suggests a role for the complex in membrane scission that is needed for sequestration of fragments of the ER or nucleus within autophagosomes.

The endoplasmic reticulum (ER) spreads throughout the cytoplasm as one continuous structure that nonetheless contains structurally distinct regions. In yeast, these include a polygonal network of tubules and fenestrated sheets tethered to the plasma membrane, termed the cortical ER. Several cytoplasmic ER sheets connect the cortical ER to the outer nuclear membrane (ONM) (1). The ER is not only continuous with the ONM, the two membranes overlap extensively in both composition and function.

More than a third of the proteome enters the lumen of the ER and nuclear envelope (NE) as nascent polypeptides, which must undergo folding prior to export by vesicular transport to other cellular destinations (2). Protein misfolding (3) can lead to the accumulation of protein aggregates or condensates within the lumen (4) that impair essential ER and nuclear functions. Segments of the ER and nucleus undergo autophagosome-mediated degradation, termed ER-phagy and nucleophagy, to remove damaged regions and misfolded proteins, or to recycle unneeded cellular components in response to nutrient deprivation (5). ER-phagy is also required for ER remodeling during aging and for mTOR-dependent lifespan extension (6).

Autophagosome-mediated degradation can be either nonselective (bulk autophagy) or selective (7). Selective autophagy leads to the degradation of a specific target, such as a domain of the ER or nucleus and relies on selective autophagy receptors (8–10). The receptor associates with a target and links it to the autophagic machinery by binding to Atg8, a lipidated protein on the membrane of the autophagosomal precursor (11–13).

Since the ER and NE forms one continuous structure with many essential functions, they cannot be digested whole, rather segments of these organelles must first be released by membrane scission to allow their sequestration within an autophagosome (8, 9). Atg40, the selective ER-phagy receptor in budding yeast, contains, in addition to its Atg8 interacting motif (AIM), a reticulon homology domain that inserts into the ER membrane, adding positive curvature, which facilitates membrane scission (11). In fission yeast, these two functions are divided between two components; Epr1 is a selective AIM-containing receptor, while Yep1 adds positive membrane curvature (14, 15). The NE presents a particularly complex challenge for the autophagy machinery as it comprises two membranes, the inner nuclear membrane (INM) and ONM, both of which are subject to autophagic degradation. In budding yeast, Atg39, a selective nucleophagy receptor, spans the ONM. Its luminal carboxy terminal domain contains three amphipathic helices that bind to the luminal surface of the INM, while its cytosolic amino terminal domain contains an AIM that promotes association with an autophagosomal precursor as well as a binding region for Atg11 (11, 16–18). Atg11 is a scaffold protein that recruits the components needed for autophagosome assembly. Through these Atg39-mediated interactions, segments of both the INM and ONM are sequestered together in a concerted reaction as a double membrane, nuclear-derived vesicle within an autophagosome.

Beyond the selective autophagy receptors, additional components have been implicated in different aspects of cortical ER-phagy. Cargo must be actively coupled to the ER autophagy receptor (19). A coat complex called Lst1-Sec23 in yeast or SEC24C-SEC23 in mammals, binds to the receptor and acts to capture ER membrane proteins and misfolded proteins as cargo within the targeted domain (19–21). In the absence of Lst1/SEC24C, the receptor is delivered to the vacuole/lysosome without cargo (19). Three-way junctions in the ER network are major sites of ER-phagy and the loss of Lnp1, a conserved protein that stabilizes these junctions, leads to an ER-phagy defect in yeast and mammalian cells (22). Additionally, the cortical ER must be physically displaced from the plasma membrane in yeast so that it can associate with the autophagy machinery. This displacement requires actin assembly at sites of interaction between the cortical ER and endocytic pits on the plasma membrane. The cortical ER is linked to endocytic pits by a chain of interactions including Scs2/Scs22, Osh6/Osh7, and Myo3/Myo5. Loss of these components blocks displacement of the cortical ER and impairs ER-phagy (23). It is not yet clear how many additional components are required for ER-phagy or nucleophagy.

Continuities between the INM and ONM exist at distinct points, creating aqueous pores that allow transport between the nucleoplasm and cytoplasm. During interphase, or in organisms such as yeast that undergo closed mitosis, de novo formation of a nuclear pore requires fusion of the INM and ONM at the site of pore assembly. Several reports have implicated a complex of Apq12, Brl1, and Brr6 in this membrane fusion event in yeast (24–27). All three of these proteins span the ER or nuclear membrane twice, with both the amino and carboxy termini facing the cytoplasm (or nucleoplasm). The two transmembrane domains flank a luminal region containing an amphipathic helix (25, 26, 28). These proteins are thought to work together to direct fusion of the ONM and INM by acting across the lumen of the NE to interact with the opposing membrane (28, 29). Here, we report the unexpected finding that the Apq12/Brr6/Brl1 complex is required for both Atg39-dependent turnover of the NE and Atg40-dependent turnover of the ER. We present evidence to support the proposal that Apq12 works in concert with the receptors to remodel the NE during nucleophagy, and the ER during ER-phagy.

## Results

### Apq12 Is Needed for ER-Phagy and Nucleophagy.

**Fig. 1.**
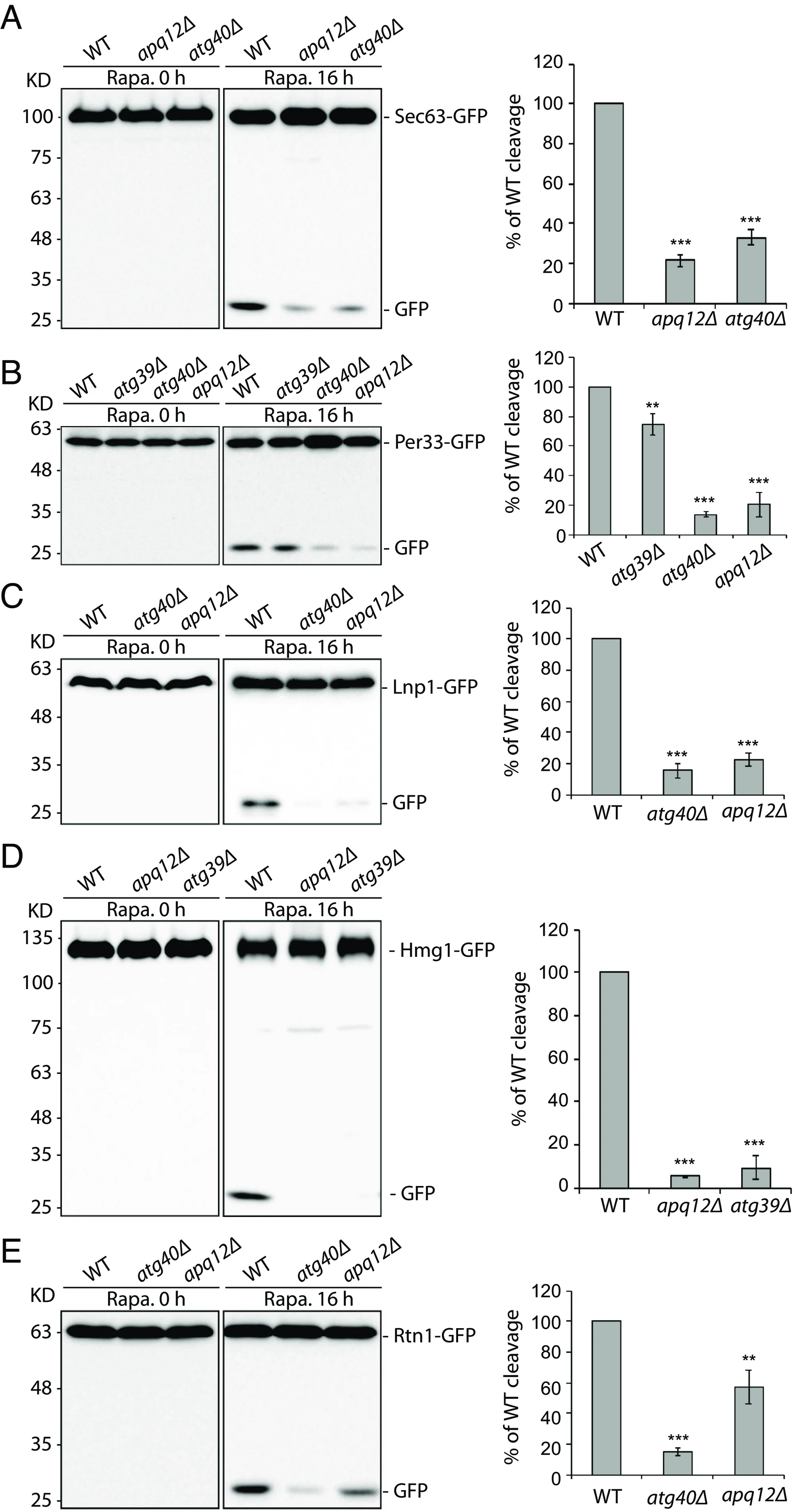
Apq12 is required for both ER-phagy and nucleophagy. Yeast cells expressing the indicated GFP-tagged proteins were harvested before and after 16 h of rapamycin treatment, and GFP cleavage was analyzed by western blotting using anti-GFP antibodies (*Left*). The percentage of free GFP relative to total GFP was quantified for the 16 h rapamycin-treated samples (*Right*), with WT set to 100% and all mutants normalized to the WT control. Error bars represent SD (N = 3 independent experiments). *P* values were calculated by comparing each mutant with WT using Student’s *t* test: **P < 0.01; ***P < 0.001. Panels show cleavage of (*A*) Sec63-GFP, (*B*) Per33-GFP, (*C*) Lnp1-GFP, (*D*) Hmg1-GFP, and (*E*) Rtn1-GFP.

To identify components involved in ER-phagy, we conducted a microscopy-based screen of the yeast deletion library for mutants exhibiting defects in delivery of the ER marker, Sec61-GFP, to the vacuole following autophagy induction with the TOR inhibitor, rapamycin (30). The *apq12Δ* strain was among the candidates identified. Since Sec61 is found on both the ER and ONM, to clarify the role of Apq12 we evaluated the efficiency of autophagic turnover of various green fluorescent protein (GFP)-tagged proteins marking either both structures or selectively marking just one. Upon delivery to the vacuole, GFP fusion proteins are cleaved, yielding free GFP which is resistant to further degradation (31). Sec63-GFP is present on all elements of the ER as well as the ONM. The *apq12Δ* mutant exhibited a dramatic decrease, relative to wild-type (WT), in cleavage of Sec63-GFP to free GFP after rapamycin treatment (Fig. 1*A*). This decrease was comparable to that observed in cells deleted for the selective ER-phagy receptor, Atg40. Fluorescence microscopy confirmed the delivery of Sec63-GFP to the vacuole in WT cells, but not in *apq12Δ* or *atg40Δ* cells, (*SI Appendix*, Fig. S1 *A* and *C*) following rapamycin treatment.

To extend this observation with other proteins, we examined Per33 and Lnp1. Per33-GFP, like Sec63, is found on all elements of the ER and the ONM. The *apq12Δ* strain exhibited a block in rapamycin-induced cleavage of Per33-GFP that was comparable to that of the *atg40Δ* strain (Fig. 1*B*). Since most of Per33 is on the ER rather than the ONM, deletion of the nucleophagy receptor, Atg39, had a relatively small effect (Fig. 1*B*). Lnp1 localizes specifically to the site where two tubules fuse, the three-way junctions in the cortical ER (32). The *apq12Δ* mutant exhibited a dramatic decrease, relative to WT, in cleavage of Lnp1-GFP to free GFP after rapamycin treatment (Fig. 1*C*). This decrease was comparable to that observed in cells deleted for Atg40.

To evaluate the role of Apq12 in nucleophagy, we examined Hmg1-GFP, which exclusively marks the ONM. Cleavage of Hmg1-GFP was almost completely blocked in *apq12Δ* mutant cells, an inhibition similar in severity to that seen upon loss of the nucleophagy receptor, Atg39 (Fig. 1*D*). Fluorescence microscopy confirmed the delivery of Hmg1-GFP to the vacuole in WT cells, but not in *apq12Δ* or *atg39Δ* cells (*SI Appendix*, Fig. S1 *B* and *D*), following rapamycin treatment. Thus, the Apq12 requirement in autophagy extends to an ONM protein.

Due to its preference for highly curved membranes the reticulon protein, Rtn1, localizes to cortical ER tubules and the edges of ER sheets, but is absent from the ONM (33, 34). Cleavage of Rtn1-GFP was partially inhibited in the *apq12Δ* mutant, whereas the *atg40Δ* mutant exhibited a more extensive cleavage defect (Fig. 1*E*). The partial inhibition of Rtn1-GFP cleavage by *apq12Δ* is consistent with the possibility of multiple pathways, only one of which requires Apq12. As prior studies have shown that loss of the yeast VAP homologs, Scs2 and Scs22, leads to a partial defect in Rtn1-GFP cleavage (35), we asked if Apq12 and Scs2/22 act on the same or different pathways. We compared Rtn1-GFP cleavage in WT, *apq12Δ, scs2/22Δ,* and *apq12Δ scs2/22Δ* cells (*SI Appendix*, Fig. S1*E*). Averaging three measurements showed that, relative to WT, the *apq12Δ* strain was inhibited by 27%, the *scs2/22Δ* strain was inhibited by 34% and the *apq12Δ scs2/22Δ* strain was inhibited by 54%. Thus, the *apq12Δ* and *scs2/22Δ* cleavage defects appeared to be approximately additive, suggesting that Apq12 and Scs2/22 act on different cortical ER-phagy pathways. In total, the data indicate a broad requirement for Apq12 in both ER-phagy and nucleophagy, although Rtn1 degradation appears to use both Apq12-dependent and Apq12-independent pathways.

**Fig. 2.**
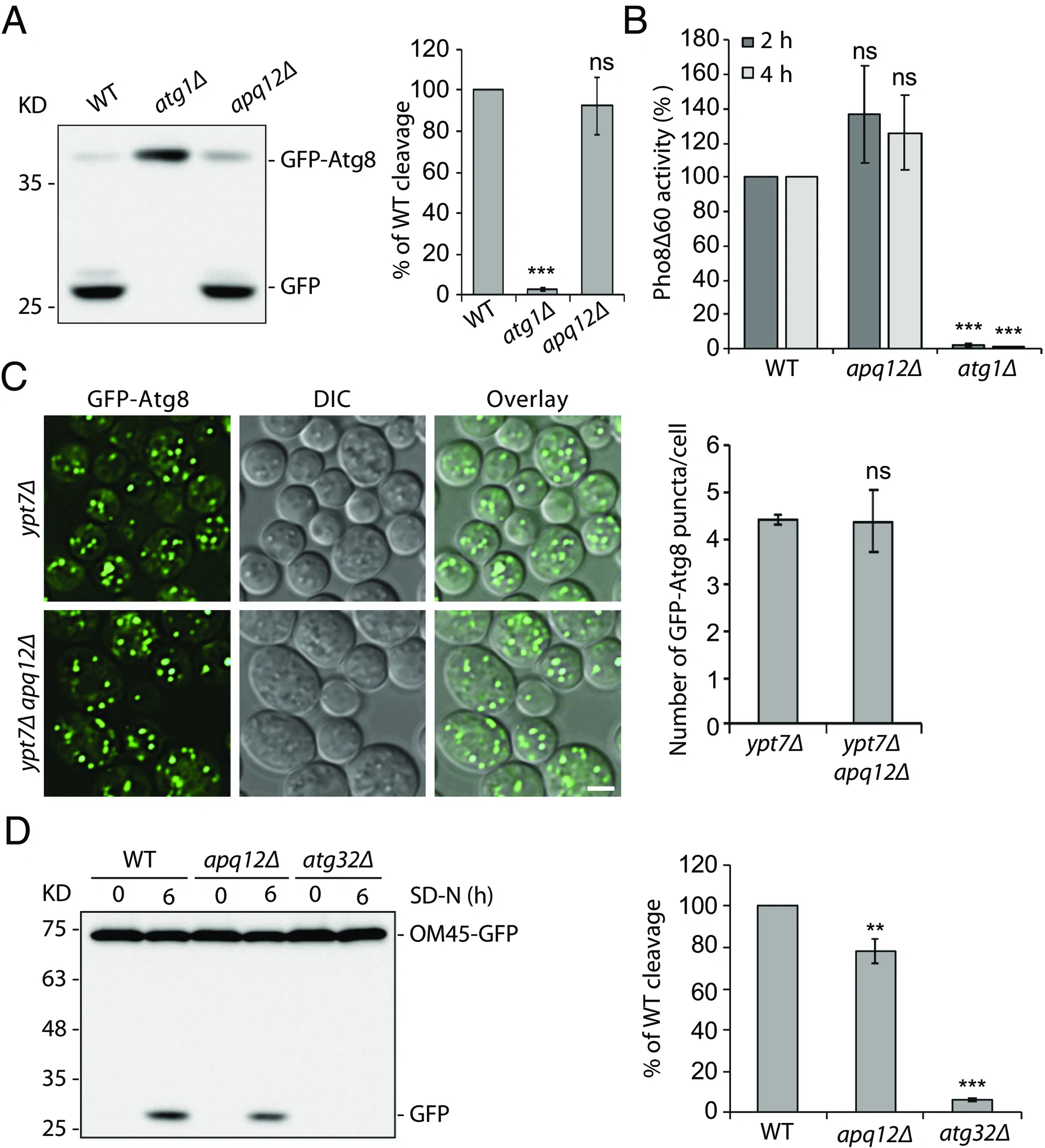
Characterization of the role of Apq12 in different autophagy pathways. (*A*) Apq12 is not required for the cleavage of GFP-Atg8. Yeast cells expressing GFP-Atg8 were treated with rapamycin for 16 h, and lysates were analyzed by western blotting using anti-GFP antibodies (*Left*). The percentage of free GFP relative to total GFP was quantified (*Right*), with WT set at 100% and mutants normalized accordingly. Error bars indicate SD (N = 3 independent experiments). *P* values: ns, not significant, *P* ≥ 0.05; **P < 0.01; ***P < 0.001 (Student’s *t* test). (*B*) ALP activity in cells expressing Pho8Δ60 was measured as described in *Materials and Methods*. Autophagy was induced by nitrogen starvation for 2 h and 4 h. Activity in each mutant was normalized to WT (100%). Statistical analysis was the same as in (*A*). (*C*) Apq12 is not required for autophagosome assembly. (*Left*) Yeast cells expressing GFP-Atg8 were treated with rapamycin for 16 h and then imaged (Scale bar, 2 μm in all panels.) (*Right*) GFP-Atg8 puncta per cell were quantified (~200 cells per experiment). Error bars represent SD (N = 3 independent experiments). *P* values were calculated by comparing the double deletion (*apq12Δ ypt7Δ*) with the single deletion (*ypt7Δ*) using Student’s *t* test: ns, not significant, *P* ≥ 0.05. (*D*) Apq12 is not required for mitophagy. OM45-GFP cleavage was analyzed by western blotting before and after 6 h of nitrogen starvation (*Left*). Quantification and statistical analysis (*Right*) were performed as in (*A*).

We next addressed if the ER-phagy and nucleophagy defects observed in the *apq12Δ* strain reflect a general defect in autophagic flux or autophagosome assembly. To quantify autophagic flux we measured cleavage of the autophagosome marker, GFP-Atg8 to free GFP. We observed no significant decrease in GFP-Atg8 cleavage in *apq12Δ* cells relative to WT after 16 h of rapamycin treatment (Fig. 2*A*), indicating that autophagic flux to the vacuole is independent of Apq12, while loss of Atg1, a component needed for autophagy induction, blocked cleavage. At early time points, there was a minor inhibition of GFP-Atg8 cleavage in *apq12Δ* cells relative to WT (*SI Appendix*, Fig. S2*A*). Fluorescence microscopy confirmed vacuolar delivery of GFP-Atg8 in WT and *apq12Δ* cells, but not *atg1*Δ cells (*SI Appendix*, Fig. S2*B*). As a further test of autophagosome function, bulk autophagy was assayed in WT, *apq12*Δ and *atg1*Δ cells by measuring alkaline phosphatase activity using the Pho8Δ60 reporter. This construct expresses an autoinhibited form of phosphatase in the cytosol. Upon delivery of an aliquot of cytosol to the vacuole by bulk autophagy, the autoinhibitory domain of Pho8Δ60 is cleaved, forming enzymatically active phosphatase, which can be readily measured using a colorimetric assay (36). After either 2 or 4 h of autophagy induction by nitrogen starvation (SD-N), the *apq12*Δ strain yielded phosphatase activity levels that were not significantly different from WT, while *atg1*Δ cells were strongly inhibited (Fig. 2*B*). To measure autophagosome assembly, we counted the number of GFP-Atg8 puncta in *apq12Δ ypt7Δ* cells relative to *ypt7Δ* cells following treatment with rapamycin. Since Ypt7 is required for fusion of mature autophagosomes with the vacuole (37), counting the number of GFP-Atg8 puncta per cell in a *ypt7Δ* background yields a direct measure of autophagosome assembly. We observed no significant change in rapamycin-induced autophagosome assembly in *apq12Δ ypt7Δ* cells relative to *ypt7Δ* cells (Fig. 2*C*). In total, these results indicate that Apq12 is not required for autophagic flux or for autophagosome assembly.

To determine if Apq12 functions on a different selective autophagy pathway we examined the cleavage of a mitochondrial outer membrane marker, OM45-GFP (38). Following induction of mitophagy by nitrogen starvation (SD-N) we observed only a minor defect in OM45-GFP cleavage in *apq12Δ* cells relative to WT (Fig. 2*D*). In contrast, loss of the selective mitophagy receptor, Atg32, led to a dramatic inhibition. In total, these results suggest that Apq12 acts primarily in ER-phagy and nucleophagy, a finding consistent with the localization of Apq12 on ER and nuclear membranes (24).

**Fig. 3.**
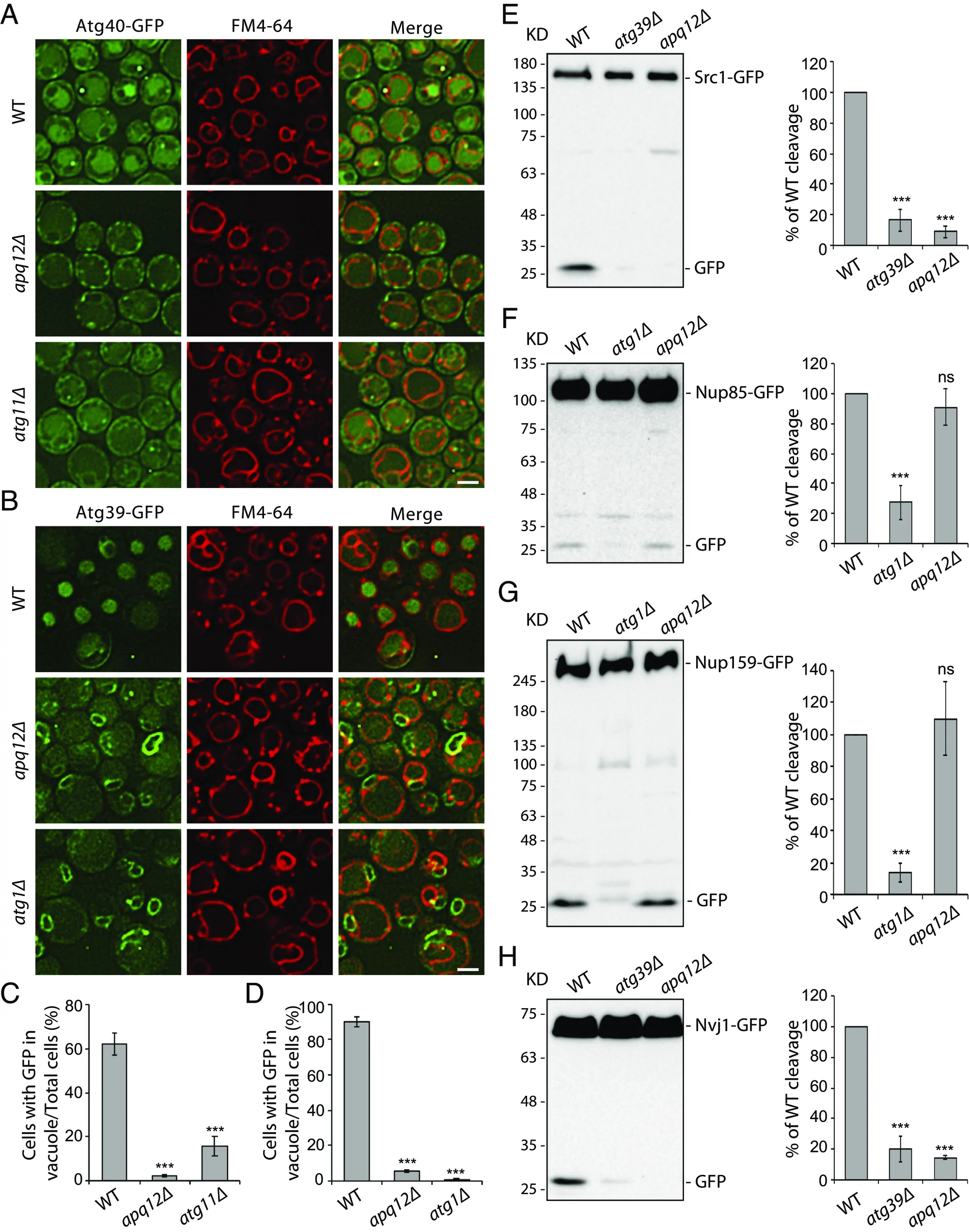
Apq12 is required for autophagic degradation of Atg40, Atg39, and its cargo. (*A*) Yeast cells (WT, *apq12Δ*, and *atg11Δ*) expressing Atg40-GFP were treated with rapamycin for 16 h and then imaged after FM4-64 staining of vacuole membranes. (Scale bar, 2 μm in all panels.) (*B*) Yeast cells (WT, *apq12Δ*, and *atg1Δ*) expressing Atg39-GFP were treated with rapamycin for 16 h and then imaged after FM4-64 staining of vacuole membranes. (Scale bar, 2 μm in all panels.) (*C*) The percentage of Atg40-GFP–expressing cells showing GFP in the vacuole was quantified. *P* values were calculated by comparing each mutant with WT using Student’s *t* test: Error bars represent SD, n = 3 independent experiments. ***P < 0.001. (*D*) The percentage of Atg39-GFP–expressing cells showing GFP signal in the vacuole was quantified and statistically analyzed as in (*C*). (*E*–*H*) GFP cleavage was analyzed in cells expressing different GFP-tagged proteins after 16 h rapamycin treatment (*E*, Src1-GFP; *F*, Nup85-GFP; *G*, Nup159-GFP; *H*, Nvj1-GFP). Cell lysates were examined by western blotting using an anti-GFP antibody (*Left*) and the percentage of free GFP relative to total GFP was quantified (*Right*), with WT set to 100% and all mutants normalized to the WT control. Error bars represent SD (N = 3 independent experiments). *P* values were calculated by comparing each mutant with WT using Student’s *t* test: ns, not significant, *P* ≥ 0.05; ***P < 0.001. See *SI Appendix*, Fig. S3 (*C*–*F*) for western blot analysis of samples taken prior to treatment with rapamycin corresponding to panels (*E*–*H*).

We assessed the vacuolar delivery and autophagic degradation of the ER-phagy receptor, Atg40-GFP, and the nucleophagy receptor, Atg39-GFP. Fluorescence microscopy demonstrated delivery of Atg40-GFP to the vacuole in WT cells, but not in *apq12Δ* or *atg11Δ* cells after rapamycin treatment (Fig. 3 *A* and *C*). Cleavage of Atg40-GFP was strongly inhibited in *apq12Δ* cells relative to WT after rapamycin treatment, indicating a defect in the ER-phagy receptor delivery pathway that was comparable to the loss of Atg11 (*SI Appendix*, Fig. S3*A*). Atg39-GFP was imaged in WT and *apq12Δ* cells after treatment with rapamycin (Fig. 3 *B* and *D*). Atg39-GFP exclusively labeled the vacuole in WT cells, while in *apq12Δ* cells the NE and associated puncta were labeled but the vacuole was not. Prior to starvation, endogenous expression of Atg39-GFP was undetectable by immunoblot analysis (*SI Appendix*, Fig. S3*B*). Upon nitrogen starvation, Atg39-GFP synthesis was induced. Rapid delivery to the vacuole in WT cells led to efficient processing to free GFP, allowing detection of only a trace of the full-length fusion protein after 6 h of starvation. In contrast, very little free GFP was detected in *apq12Δ* cells and a doublet representing the full-length Atg39-GFP fusion was observed at 6 h and 24 h of nitrogen starvation (*SI Appendix*, Fig. S3*B*). The appearance of full-length Atg39-GFP as a doublet, somewhat higher in molecular weight than predicted by sequence has been previously reported (11). Interestingly, in that study, when eight residues in the Atg11 binding region of Atg39 were replaced with Ala, the band appeared as a lower molecular weight singlet, suggesting a posttranslational modification. Together these results indicate that Apq12 is required for vacuolar delivery of both Atg40 and Atg39 following autophagy induction.

### Apq12 Is Required for Autophagy of INM and ONM, but Not Nuclear Pore Proteins.

The NE consists of several distinct domains which differ in their use of selective autophagy receptors. We explored which domains require Apq12 function for their degradation. During Atg39-mediated nucleophagy, segments of both the INM and ONM are sequestered together as a double membrane, nuclear-derived vesicle within an autophagosome (16–18, 39). To assess the role of Apq12 in autophagic turnover of the INM, we measured cleavage of the INM protein, Src1-GFP (also called Heh1). Cleavage was severely inhibited in both *apq12Δ* cells and *atg39Δ* cells relative to WT after rapamycin treatment (Fig. 3*E*, see *SI Appendix*, Fig. S3*C* for 0 h samples), confirming that Apq12, like Atg39, is required for autophagy of both INM and ONM proteins.

Nuclear pore components undergo autophagic degradation (NPC-phagy) by a pathway that is independent of Atg39 and instead uses Nup159 as a selective NPC-phagy receptor (40, 41). The efficiency of cleavage of the outer ring protein, Nup85-GFP, was similar in WT and *apq12Δ* cells following treatment with rapamycin, while loss of Atg1 largely blocked cleavage (Fig. 3*F*, see *SI Appendix*, Fig. S3*D* for 0 h samples). The efficiency of cleavage of Nup159-GFP was also similar in WT and *apq12Δ* cells following treatment with rapamycin, while loss of *ATG1* blocked cleavage (Fig. 3*G*, see *SI Appendix*, Fig. S3*E* for 0 h samples). These data indicate that NPC-phagy is independent of Apq12, just as it was previously shown to be independent of Atg39 (40).

The nuclear–vacuole junction constitutes a distinct domain of the ONM formed by the interaction of Nvj1 on the ONM, with Vac8 on the vacuole. Autophagic degradation of Nvj1 has been shown to require Atg39 function (42). We found that cleavage of Nvj1-GFP was blocked in *apq12Δ* cells relative to WT following treatment with rapamycin, as it was in *atg39Δ* cells (Fig. 3*H*, see *SI Appendix*, Fig. S3*F* for 0 h samples). In total, the pattern of requirements for Apq12 in autophagic turnover of different domains of the NE matches the pattern of requirements for Atg39; INM and ONM proteins require both Apq12 and Atg39, while NPCs require neither Apq12 nor Atg39. This supports the conclusion that Apq12 acts on the Atg39-dependent nucleophagy pathway.

### Apq12 Works with Brl1 and Brr6 on a Pathway Independent of Nuclear Pore Assembly.

**Fig. 4.**
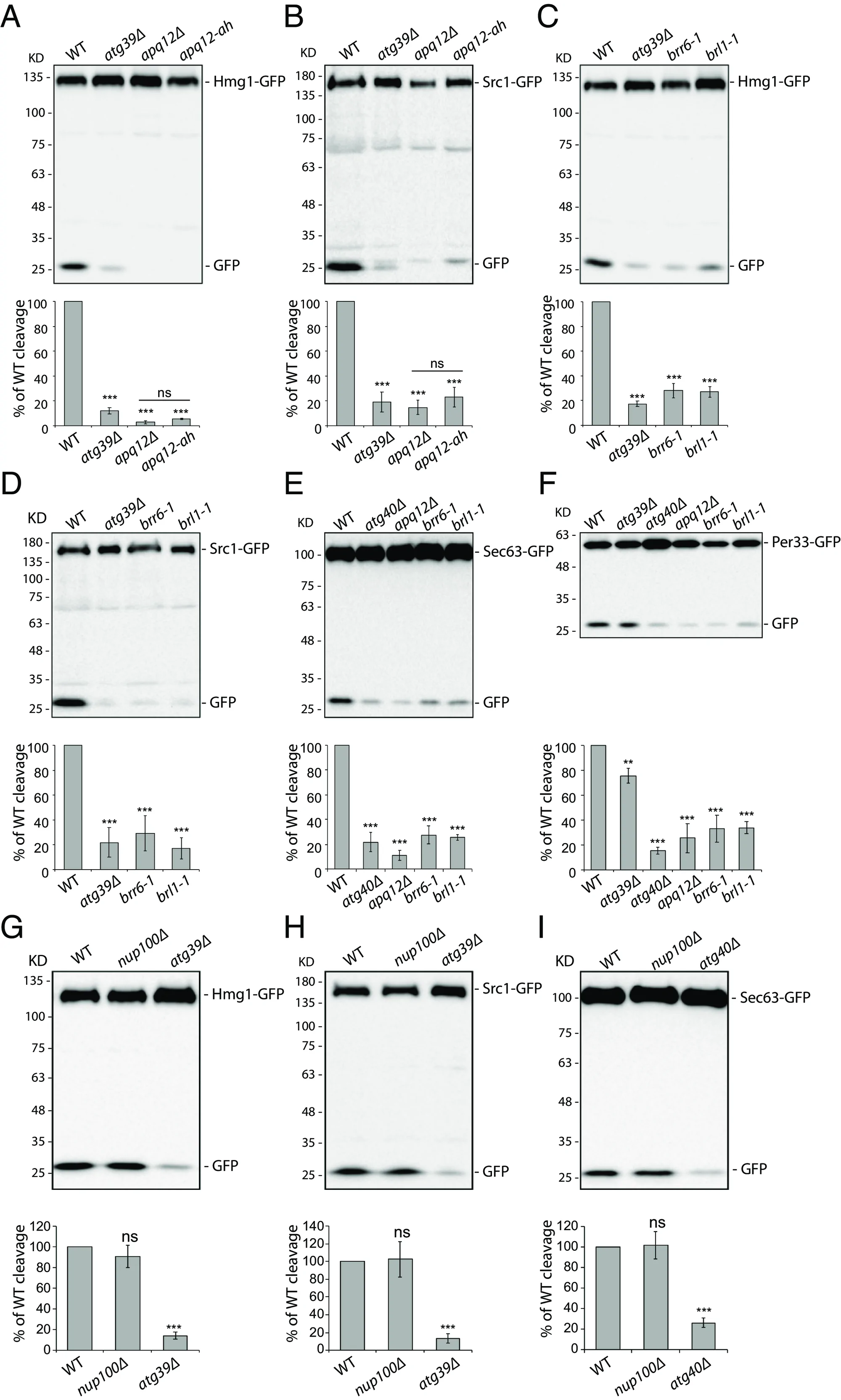
Apq12 works with Brl1 and Brr6 on a pathway independent of nuclear pore assembly. Yeast cells expressing the indicated GFP-tagged proteins were treated with rapamycin for 16 h, and GFP cleavage was analyzed by western blotting using an anti-GFP antibody (*Upper*). The percentage of free GFP relative to total GFP was quantified (*Lower*), with WT set to 100% and all mutants normalized to the WT control. Error bars represent SD (N = 3 independent experiments). *P* values were calculated by comparing each mutant with WT using Student’s *t* test: ns, not significant (*P* ≥ 0.05); **P < 0.01; ***P < 0.001. Panels show cleavage of the following GFP fusion proteins: (*A*) Hmg1-GFP, (*B*) Src1-GFP, (*C*) Hmg1-GFP, (*D*) Src1-GFP, (*E*) Sec63-GFP, (*F*) Per33-GFP, (*G*) Hmg1-GFP, (*H*) Src1-GFP, and (*I*) Sec63-GFP. See *SI Appendix*, Fig. S4 for western blot analysis of samples taken prior to treatment with rapamycin.

Apq12 has been shown to facilitate nuclear pore formation, possibly by catalyzing the fusion of the INM and ONM during its transient association with nascent pores (24, 25, 43). *APQ12* encodes a 12.5 kD protein with two transmembrane domains flanking a short helical domain that resides within the lumen of the NE or ER. The luminal helix is predicted to be amphipathic in nature and an allele with mutations within this helix (*apq12-ah*) that reduces its amphipathic nature has been shown to impair the function of Apq12 in nuclear pore formation (25). We tested the effect of the *apq12-ah* allele on rapamycin-induced cleavage of the ONM protein Hmg1-GFP and the INM protein Src1-GFP. Cleavage of both proteins was severely blocked in the *apq12-ah* strain relative to the WT strain (Fig. 4 *A* and *B*, see *SI Appendix*, Fig. S4 *A* and *B* for 0 h samples), implying that the amphipathic nature of the Apq12 luminal helix is critical for its role in nucleophagy.

Apq12 promotes nuclear pore formation in concert with Brl1 and Brr6 (24) and all three proteins coprecipitate as a complex. We therefore determined if Brl1 and Brr6 also act in ER-phagy and nucleophagy. Since *BRL1* and *BRR6* are essential genes, disruptions of these genes are not present in the deletion library and consequently were not examined in our initial screen. We therefore tested the effects of conditionally lethal alleles of *brl1* and *brl6* on the autophagic cleavage of several nuclear membrane and ER proteins. The *brl1-1* allele blocks growth at 37 °C, while *brr6-1* slows growth below 25 °C. Rapamycin-induced cleavage of Hmg1-GFP, Src1-GFP, Sec63-GFP, and Per33-GFP was strongly inhibited in *brl1-1* and *brr6-1* cells at 30 °C, a growth permissive temperature for both mutants (Fig. 4 *C*–*F* respectively, see *SI Appendix*, Fig. S4 *C–F* for 0 h samples). These blocks were not due to a general defect in autophagic flux, as the rapamycin induced cleavage of GFP-Atg8 was not significantly affected in either *brl1-1* or *brr6-1* cells (*SI Appendix*, Fig. S4*G*). These results suggest that Apq12 works in concert with Brr6 and Brl1 to promote ER-phagy and nucleophagy. Consistent with these roles, Brr6 and Brl1 are paralogous proteins that are found in both the ER and NE membranes, although in contrast to Apq12, they exhibit a higher concentration on the NE relative to the cortical ER (24).

Since the Apq12/Brr6/Brl1 complex is known to facilitate the formation of new nuclear pores, we determined if deletion of a known NPC component would inhibit ER-phagy and nucleophagy, phenocopying the *apq12Δ*, *brl1-1* and *brr6-1* mutants. We chose to analyze a *nup100Δ* strain since it is viable due to partial redundancy with *NUP116,* yet it still impacts nuclear transport (44). No significant effect was observed on cleavage of Hmg1-GFP, Src1-GFP, or Sec63-GFP in the *nup100Δ* strain after rapamycin treatment (Fig. 4 *G*–*I* respectively, see *SI Appendix*, Fig. S4 *H*–*J* for 0 h samples). Therefore, the effects of *apq12Δ, brl1-1* and *brr6-1* on ER-phagy and nucleophagy do not appear to be a downstream consequence of their effects on nuclear transport. Rather, the Apq12/Brr6/Brl1 complex likely plays roles in ER-phagy and nucleophagy that are independent of its role in nuclear pore formation.

### Nucleophagy and ER-Phagy Defects in *apq12Δ* Do Not Correlate with Changes in Lipid Composition or ER Structure.

Loss of Apq12 has been found to affect lipid composition in a temperature-dependent fashion. At 16 °C, *apq12Δ* cells exhibit an increase in storage lipids with a corresponding decrease in glycerol phospholipids; however, the effects are less apparent at 30 °C, and at 37 °C lipid composition is nearly normal (25). Since our studies were done at 30 °C, we compared cleavage of Hmg1-GFP in *apq12Δ* and WT at 30 °C, 35 °C, and 37 °C. Cleavage was found to be efficiently blocked in *apq12Δ* cells at all three temperatures, (*SI Appendix*, Fig. S4*K*) indicating that the nucleophagy defect does not correlate with changes in lipid composition.

We also asked if the loss of Apq12 function leads to a major alteration in the architecture of the ER that could potentially underlie the observed ER-phagy defect. We examined the localization of Rtn1-mCherry, marking ER tubules and the edges of ER sheets and Lnp1-3xEGFP, marking a subset of three-way ER network junctions. We focused on the peripheral ER where structural details are most evident and observed no obvious difference in the architecture of the ER in *apq12Δ* cells relative to WT cells, either before or after 3 h of treatment with rapamycin or after growth in SD-N for 2 h (*SI Appendix*, Fig. S4*L*).

### Ordering the *apq12Δ* Block with Respect to Nucleophagy and ER-Phagy Landmarks.

The Atg39-dependent nucleophagy pathway has been defined at a morphological level (16–18, 39). Following induction of autophagy, Atg39 synthesis increases and Atg39 puncta form on the ONM, typically at the edge of a nuclear vacuolar junction. These puncta extend into the cytoplasm, and an INM marker protein is often observed within their luminal space (16–18). The Atg39-marked nuclear membrane extensions associate with Atg8, a marker of the autophagosomal precursor. Scission at the base of the ONM extension allows sequestration of a double membrane, nuclear-derived vesicle within an autophagosome. Fusion of the autophagosome with the vacuole then leads to degradation of the enclosed nuclear components.

**Fig. 5.**
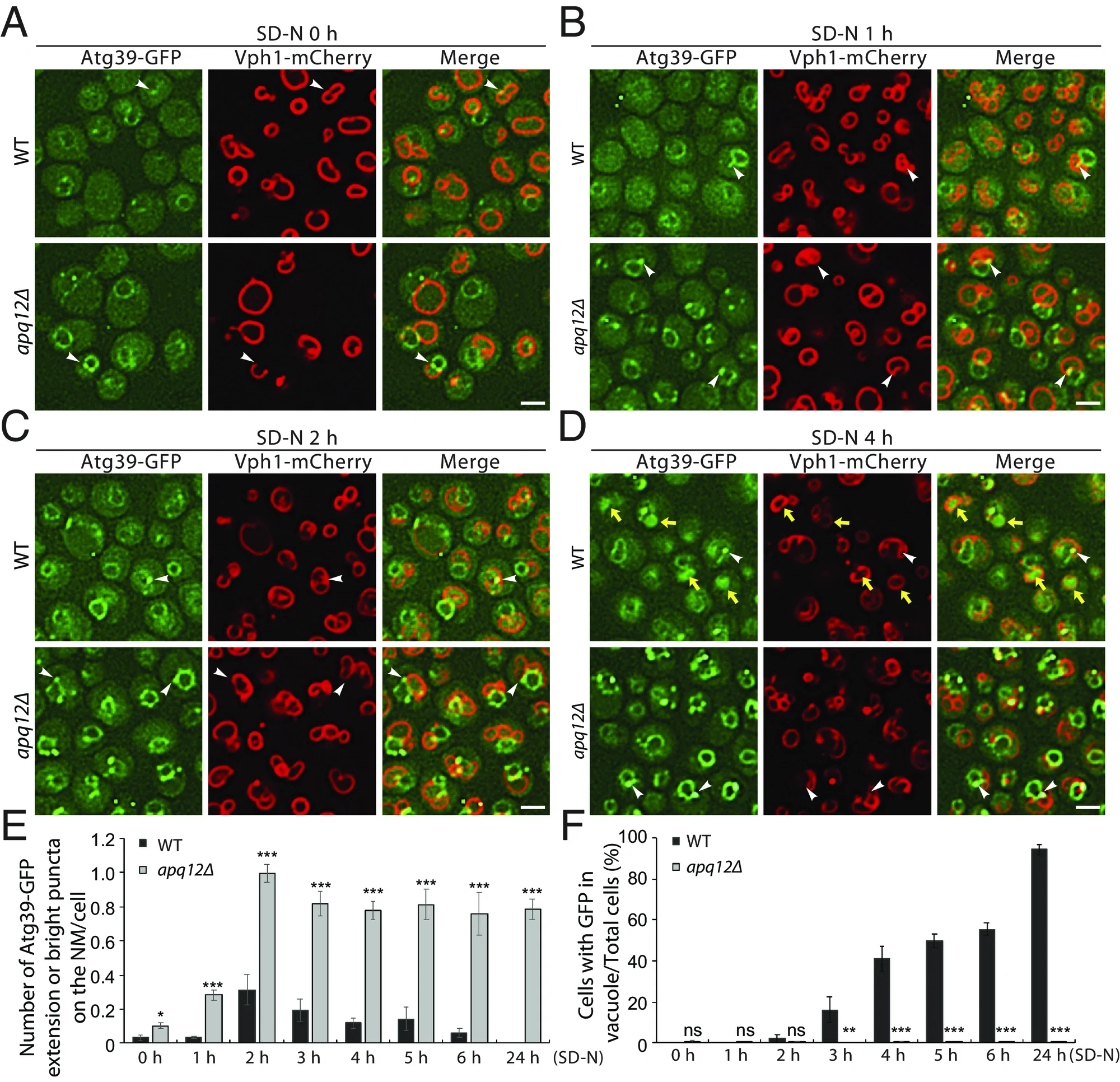
Atg39-GFP extensions and bright puncta on the nuclear membrane accumulate in *apq12Δ* cells. (*A*–*D*) Fluorescence images of WT and *apq12Δ* cells coexpressing Atg39-GFP and Vph1-mCherry before (SD-N 0 h) and after nitrogen starvation for 1 h, 2 h, and 4 h. Arrowheads indicate Atg39-GFP extensions from the nuclear membrane into the cytoplasm or bright puncta on the nuclear membrane, whereas arrows indicate Atg39-GFP signal within the vacuole. (Scale bar, 2 μm in all panels.) Panels: (*A*) SD-N, 0 h (before starvation); (*B*) 1 h; (*C*) 2 h; (*D*) 4 h. (*E*) Atg39-GFP extensions and bright puncta on the nuclear membrane were quantified per cell at the indicated time points (≥200 cells analyzed per experiment). Error bars represent SD (N = 3 independent experiments). *P* values were calculated by comparing *apq12Δ* with WT at each time point using Student’s *t* test: ns, not significant (*P* ≥ 0.05); *P < 0.05; **P < 0.01; ***P < 0.001. (*F*) The percentage of Atg39-GFP–expressing cells showing GFP signal in the vacuole was quantified and statistically analyzed as in (*E*). See *SI Appendix*, Fig. S5 for images of cells after 3 h, 5 h, 6 h, and 24 h of nitrogen starvation.

We followed Atg39-GFP by live cell imaging to ascertain the site of the *apq12Δ* nucleophagy block with respect to key landmarks on the pathway. Under normal growth conditions Atg39-GFP, expressed from the endogenous promoter and terminator, was observed as faint fluorescence on the NE with some weak punctate concentrations in both WT and *apq12Δ* cells (Fig. 5 *A* and *E*). After 1 h of nitrogen starvation, brighter puncta were occasionally observed on the nuclear perimeter in WT cells and more commonly in *apq12Δ* cells (Fig. 5 *B* and *E*). These puncta were typically at an edge of a nuclear–vacuole junction, as seen in the merged image of the vacuole marker, Vph1-mCherry and Atg39-GFP (see arrowheads). These Atg39-GFP puncta often appeared as short extensions into the cytoplasm from the NE. This pattern of localization became more prominent at 2 h (Fig. 5 *C* and *E*). Quantification of the Atg39-GFP-labeled nuclear extensions and bright puncta showed a striking accumulation of this intermediate in *apq12Δ* cells relative to WT cells (Fig. 5*E*). By 3 h of nitrogen starvation, fluorescence was occasionally also observed within vacuoles in WT cells but not in *apq12Δ* cells (Fig. 5*F* and *SI Appendix*, Fig. S5*A*). By 4 h of nitrogen starvation, fluorescence was observed within the vacuoles of nearly half of the WT cells (see yellow arrows), with a corresponding reduction in the puncta on the NE (Fig. 5 *D*–*F*). In contrast, *apq12Δ* cells continued to display many bright puncta and extensions on the NE with no vacuolar fluorescence (Fig. 5 *D*–*F*). By 6 h of nitrogen starvation, fluorescence was largely restricted to the vacuole lumen in WT cells, yet *apq12Δ* cells displayed only nuclear membrane localization with bright puncta and no vacuolar fluorescence (Fig. 5 *E* and *F* and *SI Appendix*, Fig. S5*C*). After 24 h of nitrogen starvation, all WT cells exhibited only vacuole staining, while *apq12Δ* cells continued to exhibit only nuclear membrane localization with bright puncta and extensions (Fig. 5 *E* and *F* and *SI Appendix*, Fig. S5*D*). We conclude that Apq12 is not required for the formation of the Atg39 puncta extending from the nuclear membrane into the cytoplasm, nor is it required for the association of these puncta with nuclear vacuole junctions. However, in the absence of Apq12 these puncta and nuclear membrane extensions, marked by Atg39-GFP, remain attached to the NE and are not delivered to the vacuole.

**Fig. 6.**
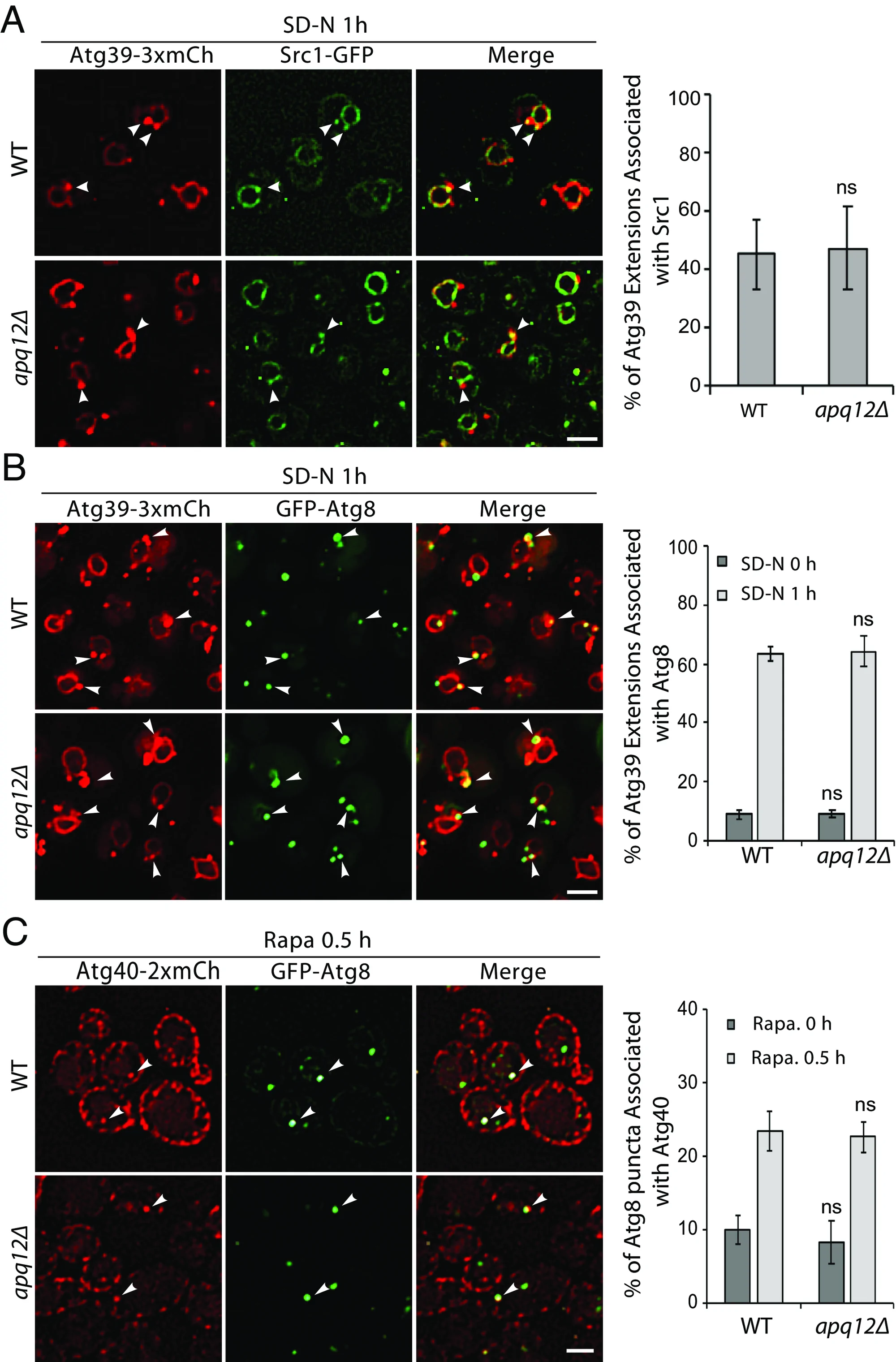
(*A*) Apq12 is not required for the inclusion of INM segments within Atg39-labeled extensions of the ONM. *Left*: Fluorescence images of WT and *apq12Δ* cells coexpressing the INM protein Src1-GFP and Atg39-3xmCherry after 1 h incubation in SD-N medium at 30 °C. Arrowheads indicate Atg39 extensions that are associated with Src1. (Scale bar, 2 μm in all panels.) *Right*: Quantification of the percentage of Atg39 extensions that are associated with Src1. Error bars represent SD (N = 3 independent experiments, at least 200 cells per experiment). *P* values were calculated by comparing *apq12Δ* with WT at each condition using Student’s *t* test: ns, not significant (*P* ≥ 0.05). (*B*) Apq12 is not required for the association of Atg39 nuclear extensions with Atg8, a marker of autophagosomes and autophagosomal precursors. (*Left*) Fluorescence images of WT and *apq12Δ* cells coexpressing Atg39-3xmCherry and GFP-Atg8 after 1 h incubation in SD-N medium at 30 °C. (Scale bar, 2 μm in all panels.) Arrowheads indicate Atg39 extensions that are associated with Atg8. (*Right*) Quantification of the percentage of Atg39 extensions that associate with Atg8 before and after 1 h incubation in SD-N medium, as described in (*A*). (*C*) Apq12 is not required for the association of Atg40 puncta with Atg8. (*Left*) Fluorescence images of WT and *apq12Δ* cells coexpressing Atg40-2xmCherry and GFP-Atg8 after rapamycin induction for 0.5 h at 30 °C. (Scale bar, 2 μm in all panels.) Arrowheads indicate Atg8 puncta that associate with Atg40. (*Right*) Quantification of the percentage of Atg8 puncta that associate with Atg40, as described in (*A*).

When Atg39-marked ONM extensions form in response to nitrogen starvation, segments of the INM are normally included within the lumen of the extensions (16–18). To determine if Apq12 is required for inclusion of INM within Atg39-labeled nuclear extensions we followed the INM marker, Src1-GFP in cells expressing Atg39-3xmCherry from the *RTN1* promoter during nitrogen starvation. In *apq12Δ* cells, after 1 h starvation, Atg39-3xmCherry formed nuclear extensions and about 45% of these were also labeled with Src1-GFP (Fig. 6*A*), a similar percentage as in WT cells. Thus, Apq12 is not required for the inclusion of INM within Atg39-labeled extensions of the ONM.

Atg39 punctate extensions on the NE normally associate with Atg8 puncta, marking an autophagosomal precursor or autophagosome, prior to their delivery to the vacuole (16–18). A similar fraction of Atg39-3xmCherry labeled nuclear membrane extensions were observed to associate with GFP-Atg8 puncta after 1 h of nitrogen starvation in *apq12Δ* and WT cells (Fig. 6*B*). Thus, Apq12 is not required for the association of Atg39 nuclear extensions with Atg8.

Following induction of autophagy, Atg40 synthesis increases and puncta form on the cortical ER (11). Some of these puncta associate with Atg8 puncta, marking autophagosomal precursors and autophagosomes, before delivery to the vacuole. We measured the percentage of GFP-Atg8 puncta that were associated with Atg40-2xmCherry after 0.5 h of rapamycin treatment. WT and *apq12Δ* cells displayed a similar level of association (Fig. 6*C*). In total, these data indicate that Atg39-dependent nucleophagy and Atg40-dependent ER-phagy pathways are blocked at a step after the receptors interact with an autophagosomal precursor.

### Apq12/Brr6/Brl1 Associates with Atg39 and Atg40.

**Fig. 7.**
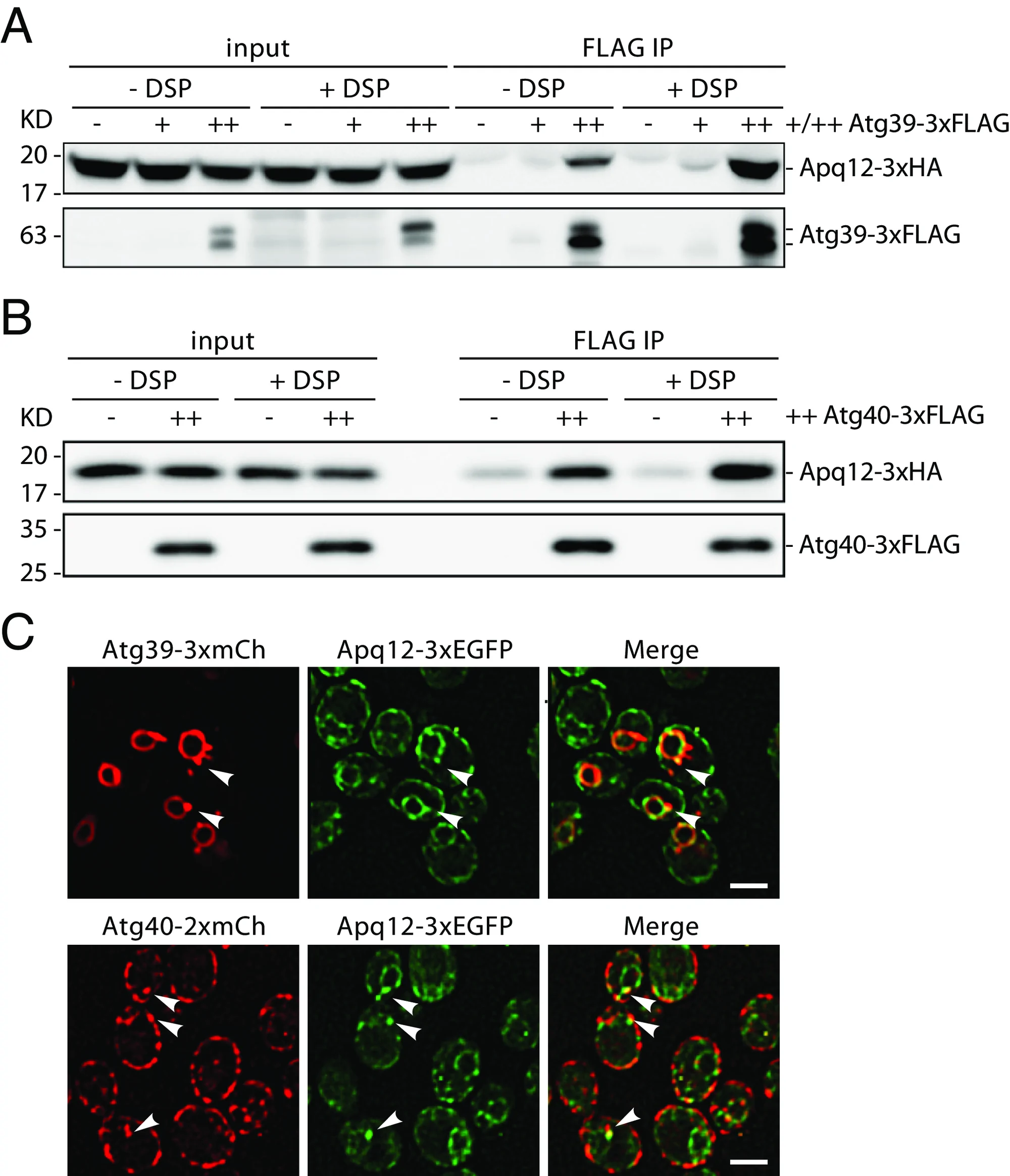
Apq12 associates with Atg39 and Atg40. (*A*) WT cells expressing Apq12-3xHA together with Atg39-3xFLAG at none (−), endogenous (+), or overexpressed level under the *CUP1* promoter (++), as indicated in the figure, were incubated with rapamycin for 3 h and then treated without (−DSP) or with (+DSP) the crosslinking reagent DSP. Cells were lysed and immunoprecipitated using anti-FLAG beads (*Materials and Methods*). Input samples and precipitates (FLAG IP) were analyzed by western blotting using an anti-HA antibody to detect Apq12-3xHA and anti-FLAG antibody to detect Atg39-3xFLAG. The input for Apq12-3xHA was 0.5%, and that for Atg39-3xFLAG was 10%. For overexpression of Atg39-3xFLAG, CuSO_4_ was added to a final concentration of 125 μM. (*B*) WT cells expressing Apq12-3xHA together with Atg40-3xFLAG at none (−) or overexpressed level under the *CUP1* promoter (++; 125 μM CuSO_4_), as indicated in the figure, were incubated with rapamycin for 3 h and then treated without (−DSP) or with (+DSP) the crosslinking reagent DSP. Cells were lysed and immunoprecipitated as described in (*A*). The input for Apq12-3xHA was 0.2%, and that for Atg40-3xFLAG was 50%. (*C*) Apq12 partially associates with Atg39 and Atg40. (*Upper*) Fluorescence images of cells coexpressing Atg39-3xmCherry and Apq12-3xEGFP after 1 h incubation in SD-N medium at 30 °C. Arrowheads indicate Atg39 extensions that associate with Apq12. (*Lower*) Fluorescence images of cells coexpressing Atg40-2xmCherry and Apq12-3xEGFP after 1 h rapamycin treatment at 30 °C. Arrowheads indicate Atg40 puncta that associate with Apq12. (Scale bar, 2 μm in all panels.)

To explore the possibility of a physical interaction between Atg39 and Apq12 we conducted coimmunoprecipitation (co-IP) assays both with and without prior treatment of intact cells with the cell permeable, reversible crosslinker dithiobis(succinimidyl proprionate) (DSP). Cells expressing Apq12-3xHA either with or without Atg39-3xFLAG, either at endogenous levels or overexpressed from the *CUP1* promoter, were treated with rapamycin for 3 h, washed, and incubated with or without DSP. The crosslinker was quenched and detergent solubilized lysates were prepared. Following precipitation with anti-FLAG antibody, the crosslinker was cleaved with dithiothreitol (DTT) and the precipitates were immunoblotted with anti-HA antibody. Without prior crosslinking, Apq12-3xHA was detected in the anti-FLAG precipitates (FLAG IP) from lysates of cells overexpressing Atg39-3xFLAG, but not from the no-FLAG control precipitates, indicating association of Apq12 with Atg39 (Fig. 7*A*). Without crosslinker no interaction was detected in cells expressing endogenous levels of Atg39-3xFLAG; however, a weak interaction was observed in the crosslinked sample, and a robust interaction was detected in cells overexpressing Atg39-3xFLAG that had been treated with crosslinker (Fig. 7*A*). These results indicate an interaction between Atg39 and Apq12 that is stabilized by treatment of intact cells with crosslinker. Interactions of Brl1-3xHA and Brr6-3xHA with overexpressed Atg39-3xFLAG were also detected (*SI Appendix*, Fig. S6 *A* and *B*, respectively), although there was some nonspecific precipitation of Brl1-3xHA and a nonspecific band (presumably an IgG light chain) ran just below Brr6-3xHA in the precipitates. Nonetheless, the data suggest that all three proteins, Apq12, Brl1, and Brr6, can associate either directly or indirectly with Atg39-3xFLAG.

We used the same protocol to look for an interaction between Atg40-3xFLAG and Apq12-3xHA. We observed a clear Apq12-3xHA signal above background in the anti-FLAG precipitates when Atg40-3xFLAG was overexpressed and no crosslinker was added (Fig. 7*B*) and the signal was increased by use of crosslinker. Thus, Atg40 interacts with Apq12, and the interaction is stabilized by treating intact cells with crosslinker. Crosslinker stabilized interactions of Brl1-3xHA and Brr6-3xHA with overexpressed Atg40-3xFLAG were also detected (*SI Appendix*, Fig. S6 *C* and *D*, respectively), although there was some nonspecific precipitation of Brl1-3xHA and a nonspecific band (presumably an IgG light chain) ran just below Brr6-3xHA in the precipitates. Thus, Apq12, Brl1, and Brr6 can also associate either directly or indirectly with Atg40-3xFLAG.

To further explore potential interactions between Apq12 and the selective autophagy receptors, we determined if Apq12-3xEGFP associates with Atg39-3xmCherry and Atg40-2xmCherry by fluorescence microscopy. Due to low levels of expression of Atg39 from the endogenous *ATG39* promoter, we used the *RTN1* promoter. Protein localization was examined after nitrogen starvation for 1 h. Apq12-3xEGFP localized to the NE and to all elements of the ER, while Atg39-3xmCherry was associated with the NE, with bright puncta, many of which appeared to extend into the cytoplasm. Many of the Atg39-3xmCherry puncta were associated with Apq12-3xEGFP (Fig. 7 *C*, *Top*). We also explored the association of Atg40-2xmCherry with Apq12-3xEGFP after 1 h of rapamycin treatment. Both proteins exhibited punctate localization on the cortical ER with some overlap (Fig. 7 *C*, *Bottom*). Apq12-3xEGFP also localized to the NE where some puncta were associated with Atg40-2xmCherry puncta near the NE (Fig. 7 *C*, *Bottom*). Thus, Apq12 partially associates with the nucleophagy and ER-phagy receptors after starvation or rapamycin treatment, consistent with the co-IP results.

Apq12 is associated with all elements of the ER as well as the NE. Degradation of Apq12 is therefore expected to depend upon the ER-phagy receptor. We found that cleavage of Apq12-GFP in response to rapamycin was largely dependent on Atg40 (*SI Appendix*, Fig. S6*E*). Brl1 is more concentrated on the nuclear membrane relative to the cortical ER. Consistent with this preferential localization we observed that cleavage of Brl1-GFP in response to rapamycin was largely dependent on Atg39 (*SI Appendix*, Fig. S6*F*).

## Discussion

We report the surprising finding that Apq12, a protein previously implicated in the formation of nuclear pores (24, 25), is required for both Atg39-dependent nucleophagy and Atg40-dependent ER-phagy. In these roles, Apq12 appears to work in conjunction with Brr6 and Brl1, two paralogous proteins that also function together with Apq12 in nuclear pore assembly (24–26, 28, 45). The roles of the Apq12/Brr6/Brl1 complex in ER-phagy and nucleophagy are probably independent of its role in nuclear pore formation since loss of Nup100, a known NPC component does not lead to ER-phagy or nucleophagy defects. Loss of Apq12 also affects lipid composition in a temperature-dependent fashion (25). However, we observed that nucleophagy was efficiently blocked in *apq12Δ* cells at temperatures where lipid composition is nearly normal, suggesting that the nucleophagy defect is not a consequence of an alteration in lipid composition. Nonetheless, we cannot exclude the possibility of a compositional change in a localized lipid pool, a change in the length or saturation of acyl chains or a change in a minor lipid species; changes that could relate to the observed autophagy defects.

The Atg39-dependent nucleophagy pathway has been well described at a morphological level (11, 16–18). Following induction of autophagy, Atg39 puncta assemble on the ONM adjacent to the nuclear vacuole junction and extend into the cytoplasm. INM-derived vesicles are observed within the ONM extensions (17). The Atg39 nuclear membrane extensions associate with an autophagosomal precursor. Scission at the base of the ONM extension allows sequestration of a double membrane nuclear-derived vesicle within an autophagosome. Fusion with the vacuole then leads to degradation of the enclosed nuclear components. Our analysis indicates that the initial stages of this pathway do not require Apq12 function. In *apq12Δ* cells Atg39 puncta form at an edge of a nuclear vacuolar junction and are frequently seen to extend into the cytoplasm while remaining attached to the ONM. An INM protein, Src1, is often observed within the extensions and Atg8 associates with the structure. Nonetheless, the Atg39 extensions fail to dissociate from the ONM, even after 24 h of starvation, leading to a dramatic accumulation of NE extensions in *apq12Δ* cells and a severe block in delivery to the vacuole. These findings suggest a role for Apq12 in scission of the ONM, although we cannot exclude a role in scission of both the INM and ONM.

The Atg40 ER-phagy pathway is topologically less complex than the Atg39 nucleophagy pathway, involving only one target membrane rather than two, nonetheless it has not been defined in as much morphological detail, due in part to slower, less synchronous kinetics. Following induction of autophagy, Atg40 forms puncta on the ER membrane network (11). Scission releases short segments of ER carrying Atg40 that are sequestered within autophagosomes and delivered to the vacuole. In *apq12Δ* cells, Atg40 puncta form and can associate with Atg8, but are blocked in delivery to the vacuole. Since *apq12Δ* cells are blocked in both the Atg39 nucleophagy pathway and the Atg40 ER-phagy pathway, Apq12 presumably plays a related role on both pathways. Given that the ER is continuous with the ONM and they share many components, the simplest explanation is that Apq12 is required for scission of both the ONM extensions bearing Atg39 and ER membrane fragments bearing Atg40. Brr6 and Brl1 appear to act in concert with Apq12 in both nucleophagy and ER-phagy.

To evaluate possible mechanistic roles for the Apq12/Brr6/Brl1 complex in ER-phagy and nucleophagy, it is instructive to review its proposed mechanism of action in nuclear pore formation. Loss of function alleles of any of these three genes lead to the formation of nuclear membrane herniations in which the INM bulges toward the ONM and early components of the nuclear pore assemble within the INM herniation (24–27). This has been interpreted as a block in fusion of the INM with the ONM at the site of pore formation. Structural modeling suggests that both Brl1 and Brr6 can form oligomeric structures composed of 8 to 20 monomers that can locally disrupt the phospholipid bilayer structure (28, 29). The luminal domains of two opposing structures are predicted to interact with each other across the lumen of the NE, potentially triggering lipid mixing that leads to membrane fusion and thus the formation of a new nuclear pore. Apq12 has a luminal domain that is too small to span the lumen, so it might play a supporting role in Brl1 and Brr6 function, consistent with the observation that it is not essential for viability, in contrast to Brl1 and Brr6, which are essential. It is not yet clear if Apq12 affects the assembly or structure of Brl1 or Brr6 oligomers; however, reduced coprecipitation of Brl1 with Brr6 was observed in *apq12Δ* cells (24).

The ability of Brr6 and Brl1 to form oligomers that locally disrupt phospholipid bilayer structure might also contribute to their postulated roles in scission of ER segments and nuclear membrane derived extensions. Instead of acting on opposing faces of the planar nuclear membranes, as they have been proposed to do in nuclear pore formation (28, 29), these structures might alternatively form collars around the base of a tubular extension emanating from either the ONM or the ER. By locally promoting lipid mixing across the lumen, such collars might catalyze tubule scission. Dynamin is known to promote tubule scission by exerting a contraction force on the cytoplasmic face of tubular membranes (17). In contrast to dynamin, we suggest that the Apq12/Brr6/Brl1 complex acts within the luminal space to catalyze lipid mixing, leading to tubule scission.

## Materials and Methods

### Strains.

The plasmids and yeast strains used in this study are summarized in *SI Appendix*, Tables S1 and S2. All single-gene deletion strains were generated following the method of Longtine et al. (46), whereas strains carrying multiple gene deletions were obtained through genetic crosses and tetrad dissection. All tagged constructs (GFP, mCherry, HA, or FLAG) were generated using integrating, 2μ, or CEN vectors.

The *brr6-1* allele carries a single point mutation (R110K) (47). A fragment corresponding to the C-terminal 450 nt of *BRR6* harboring the mutation was used to generate the integration plasmid pRS303-brr6-1-3×FLAG. The plasmid was linearized with HpaI and integrated into WT (BY4741) yeast. Correct integrants were verified by PCR and sequencing.

The *brl1-1* allele carries six point mutations (V138A, Y268C, R332S, E339G, T385A, and T406P) and four synonymous mutations at residues 82, 273, 371, and 446 (24). A fragment containing the *BRL1* promoter (437 nt), *brl1-1* (full-length *BRL1* harboring these mutations), and the *BRL1* 3′ UTR (260 nt) (gift from R. Schneiter) was cloned into pRS423 at the SacI and SalI sites. The resulting 2μ plasmid was transformed into a diploid *BRL1*/*brl1Δ* strain, since the single-gene deletion is lethal. Haploid *brl1Δ* strains carrying pRS423-brl1-1 were verified by PCR and sequencing.

The *apq12-ah* allele carries four point mutations (L58D, F61R, I62N, and F68N) (25). A fragment containing the *APQ12* promoter (656 nt), *apq12-ah* (full-length *APQ12* harboring these mutations) was cloned into pRS303-3xHA at the ApaI and PacI sites. The resulting plasmid was linearized with Bsu36I and integrated into the *APQ12* promoter region in *apq12Δ* strain. Correct integrants were verified by PCR and sequencing.

### Induction of Autophagy.

ER-phagy/nucleophagy was induced as previously described (22). Yeast cells were grown in synthetic complete or drop-out medium containing 2% glucose (SD) to an OD_600_ of 0.1 to 0.2, followed by treatment with 200 ng/mL rapamycin at 30 °C for the indicated time (0.5 to 16 h). For starvation-induced autophagy, cells were grown to an OD_600_ of 0.2 to 0.4, washed twice with sterile water, resuspended in SD-N medium (0.17% yeast nitrogen base without amino acids and ammonium sulfate, 2% glucose), and incubated at 30°C for the indicated time (1 to 24 h).

To induce mitophagy, cells expressing OM45–GFP, a mitochondrial outer membrane protein used as a mitophagy marker, were cultured in Yeast Extract–Peptone–Dextrose (YPD) media (1% yeast extract, 2% peptone, 2% glucose) to early-log phase (38). Cells were then resuspended in YPL medium to an OD_600_ of ~0.2 and incubated at 30 °C for ~24 h until reaching log phase (OD_600_ 1 to 2). Cells were subsequently washed twice with sterile water, resuspended in 5 mL SD-N medium and incubated for another 6 h.

### GFP Cleavage Assays.

GFP cleavage assays were performed as previously described (30). Following autophagy induction as described above, 5 OD_600_ equivalents of cells expressing Sec63-GFP, Per33-GFP, Lnp1-GFP, Hmg1-GFP, Rtn1-GFP, GFP-Atg8, OM45-GFP, Atg40-GFP, Atg39-GFP, Src1-GFP, Nup85-GFP, Nup159-GFP, Nvj1-GFP, Apq12-GFP, or Brl1-GFP were harvested by centrifugation at 3,000×*g* for 5 min. Cells were washed with cold water, resuspended in 1 mL of ice-cold 10% trichloroacetic acid (TCA) and incubated on ice for at least 30 min. Cells were then pelleted at 13,000×*g* for 3 min and washed twice with 1 mL of ice-cold acetone to remove residual TCA. Cell pellets were air-dried in a culture hood and resuspended in 50 μL of resuspension buffer (50 mM Tris-HCl, 1 mM EDTA, 1% SDS, 6 M urea, pH 7.5). Approximately 100 μL of silica beads were added and samples were vortexed for 5 min followed by a 5 min incubation at 55 °C in a water bath. Subsequently, 50 μL of sample buffer (150 mM Tris-HCl, 6% SDS, 6 M urea, 10% 2-mercaptoethanol, 0.002% bromophenol blue, pH 6.8) was added and samples were vortexed for 5 min and incubated at 55 °C for 10 min in a water bath. After centrifugation at 13,000×*g* for 10 min to remove debris, supernatants were subjected to SDS–PAGE. GFP-tagged fusion proteins and free GFP were detected by western blotting using an anti-GFP mouse monoclonal antibody (1:2,000 dilution, Roche, Clone 7.1 and 13.1). Images were acquired and analyzed using Bio-Rad Image Lab software. Cleavage is defined as the amount of free GFP over the total amount of free GFP plus GFP-tagged protein. The cleavage of each mutant strain was normalized to WT, which was set as 100%.

### Vacuole Staining with FM4-64.

Vacuolar membranes were stained with FM4-64 as previously described (48). Rapamycin-treated cells were collected and resuspended in 100 μL YPD medium containing FM4-64 at a final concentration of 1.6 μM. After incubation at 30 °C for 40 to 60 min, cells were pelleted and washed twice with 1 mL YPD medium. Cells were then resuspended in SC medium and mounted for imaging using an Axio Imager Z1 fluorescence microscope as described below.

### Fluorescence Microscopy.

Cells corresponding to approximately 1 OD_600_ unit were pelleted by centrifugation and imaged using an Axio Imager Z1 fluorescence microscope as previously described (23). The system was equipped with an alpha-Plan Apochromat 100×/1.46 UV VIS IR objective lens (Zeiss), a Compact Light Source HXP 120V, and an Axiocam 506 mono digital camera. Images were subsequently processed using OpenLab (Improvision), Fiji/ImageJ, or Photoshop CS4 (Adobe).

### Microscopy-Based Assay for Vacuolar Delivery.

We used a semiquantitative, microscopy-based assay to assess the vacuolar delivery of various GFP-tagged marker proteins. Vacuole membranes were labeled by either coexpressing Vph1-mCherry or by staining with FM4-64, as described above. After the indicated incubation, cells were imaged. Cell images were visually evaluated to determine if the vacuoles, defined in the red channel, were positive or negative in the green channel, using the fluorescence level of the cytoplasm as a baseline for comparison.

### Vacuolar Alkaline Phosphatase Activity.

Alkaline phosphatase (Pho8Δ60) assays were performed as previously described (22). Briefly, yeast cells grown to mid-log phase were shifted to SD-N medium for 2 or 4 h of nitrogen starvation and lysed by glass bead disruption. The resulting lysates were incubated with p-nitrophenyl phosphate substrate, and the reaction was terminated with glycine-based alkaline buffer prior to measuring absorbance at 400 nm. Enzymatic activity was normalized to total protein levels. The activity in each mutant was normalized to WT, which was defined as 100%.

### Co-IP.

Strains expressing FLAG-tagged Atg39 or Atg40 with HA-tagged Apq12, Brl1, or Brr6 were cultured at 30 °C to an OD_600_ of ~0.1. For induced Atg39-3xFLAG or Atg40-3xFLAG expression, CuSO_4_ was added to a final concentration of 125 μM, while endogenous-expressing strains were untreated. After 2 h, rapamycin was added to a final concentration of 200 ng/mL, and cells were incubated for 3 h before harvest. Cells were pelleted, washed, and resuspended in 1 mL of lysis buffer containing 2 mM DSP (25 mM Hepes pH 7.5, 150 mM NaCl, 1 mM EDTA); for samples not treated with crosslinker, DSP addition and subsequent Tris-HCl quenching were omitted. Suspensions were incubated on a nutator at 4 °C for 2 h, quenched with 100 mM Tris-HCl (pH 7.5), and incubated on ice for ~15 min before addition of protease inhibitors. Cells were lysed in 2 mL screw-cap tubes with 2 g prewashed 0.5-mm zirconia/silica beads via two 3-min bead-beating cycles with a 1-min interval on ice. Triton X-100 was added to a final concentration of 1%, and lysates were incubated at 4 °C for 15 min. Lysates were clarified by centrifugation at 20,000×*g* for 30 min, and supernatants were incubated with 10 μL anti-FLAG beads at 4 °C overnight. Beads were washed five times with 0.1% Triton X-100 in lysis buffer, eluted with Laemmli sample buffer, and analyzed by western blotting using rabbit anti-HA (Abcam, 1:2,500) or anti-FLAG (Sigma, 1:1,000) antibodies.

## Supplementary Material

Appendix 01 (PDF)

## Data Availability

Study data are included in the article and/or *SI Appendix*.
