# Supplementary material for: Atg39-dependent nucleophagy and Atg40-dependent ER-phagy require the Apq12/Brr6/Brl1 complex
Source: Proc Natl Acad Sci U S A. 2026 Sep 30;123(40):e2620353123. doi: 10.1073/pnas.2620353123 (PMC13643140; doi:10.1073/pnas.2620353123)
Supplement: Supplementary file 1 [file pnas.2620353123.sapp.pdf]

Table S1. Plasmids for yeast transformation

NRB	Alias	Plasmid name	Source
SFNB2335	SFNB2335	pRS305-Per33-GFP (LEU2, BmgBI digestion to integrate into *PER33* locus)	Ferro-Novick lab collection
NRB1711	NRB1711	pRS305-Rtn1-GFP (LEU2, HindIII digestion for integration into *RTN1* locus)	Novick lab collection
SFNB2374	SFNB2374	pRS305-Hmg1ΔN-GFP (LEU2, XbaI digestion for integration into *HMG1* locus)	Ferro-Novick lab collection
SFNB1637	SFNB1637	pRS416-GFP-Atg8 (URA3, CEN)	Ferro-Novick lab collection
NRB1708	NRB1708	pRS305-OM45-GFP (LEU2, SalI digestion for integration into *OM45* locus)	Novick lab collection
SFNB2414	SFNB2414	pRS426-pCUP1-Atg39-3xFLAG (URA3, 2μ)	Ferro-Novick lab collection
NRB1662	NRB1662	pRS426-pCUP1-Atg40-3xFLAG (URA3, 2μ)	Novick lab collection
SFNB2375	SFNB2375	pRS306-Atg40-2xmCherry (URA3, BstAPI digestion for integration into *ATG40* locus)	Ferro-Novick lab collection
NRB1714	DLP765	pRS303-Brr6-1-3xFLAG (HIS3, HpaI digestion to integrate into *BRR6* locus)	This study
NRB1715	DLP802	pRS423-Brl1-1 (HIS3, 2μ)	This study
NRB1716	DLP799	pRS303-pApq12-Apq12-ah-3xHA (HIS3, Bsu36I digestion to integrate into *APQ12* promoter locus in *apq12Δ* strain)	This study
NRB1717	SCP120	pRS305-Sec63-GFP (LEU2, PstI digestion to integrate into *SEC63* locus)	This study
NRB1718	SCP922	pRS305-Lnp1-GFP	This study
NRB1719	SCP812	pRS305-pAtg8-GFP-Atg8 (LEU2, SnaBI digestion to integrate into *ATG8* promoter locus in atg8Δ strain)	This study
NRB1720	DLP654	pRS305-Atg40-GFP (LEU2, HindIII digestion for integration into *ATG40* locus)	This study
NRB1721	DLP770	pRS305-Atg39-GFP-Atg39 3'UTR (LEU2, BamHI digestion for integration into *ATG39* locus)	This study
NRB1722	SCP670	pRS305-Src1-GFP (LEU2, SalI digestion for integration into *SRC1* locus)	This study
NRB1723	DLP776	pRS305-Nup85-GFP (LEU2, PstI digestion for integration into *NUP85* locus)	This study
NRB1724	DLP777	pRS305-Nup159-GFP (LEU2, BglII digestion for integration into *NUP159* locus)	This study
NRB1725	DLP592	pRS315-Nvj1-GFP (LEU2, CEN)	This study
NRB1726	DLP794	pRS305-Apq12-GFP (LEU2, AatII digestion for integration into *APQ12* locus)	This study
NRB1727	DLP795	pRS305-Brl1-GFP (LEU2, PstI digestion for integration into *BRL1* locus)	This study
NRB1728	DLP774	pRS305-pApq12-Apq12-3xHA (LEU2, HindIII digestion to integrate into *APQ12* promoter locus in *apq12Δ* strain)	This study
NRB1729	DLP793	pRS305-Brl1-3xHA (LEU2, PstI digestion for integration into *BRL1* locus)	This study
NRB1730	DLP779	pRS305-Brr6-3xHA (LEU2, NdeI digestion for integration into *BRR6* locus)	This study
NRB1731	DLP782	pRS306-Atg39-3xFLAG-Atg39 3'UTR (URA3, BamHI digestion for integration into *ATG39* locus)	This study
NRB1732	SCP789	pRS305-pApq12-Apq12-3xEGFP (LEU2, MscI digestion to integrate into *APQ12* promoter locus in *apq12Δ* strain)	This study
NRB1733	SCP796	pRS315-GFP-Atg8 (LEU2, CEN)	This study
NRB1734	DLP711	pRS316-pRtn1-Atg39-3xmCherry (URA3, CEN)	This study
NRB1735	SCP470	pRS306-Rtn1ΔN-mCherry (URA3, HindIII digestion for integration into *RTN1* locus)	This study
SFNB 2228	SFNB 2228	pRS305-Inp1ΔN-3xEGFP (LEU2, MscI digestion for integration into *LNP1* locus)	Ferro-Novick lab collection

Table S2. Yeast strains used in this study

NY	Alias	Genotype	Source
NY3513	SCY3443	*MAT a, his3Δ1, leu2Δ0, met15Δ0, ura3Δ0*	This study
NY3514	SCY4531	*MAT a, his3Δ1, leu2Δ0, met15Δ0, ura3Δ0 [atg39Δ::KanMX4]*	This study
NY3515	SCY4976	*MAT a, his3Δ1, leu2Δ0, met15Δ0, ura3Δ0 [atg40Δ::KanMX4]*	This study
NY3516	SCY5084	*MAT a, his3Δ1, leu2Δ0, met15Δ0, ura3Δ0 [apq12Δ::KanMX4]*	This study
NY3517	DLY1681	*MAT a, his3Δ1, leu2Δ0, met15Δ0, ura3Δ0 [brr6-1-3xFLAG::HIS3]*	This study
NY3518	DLY1812	*MAT alpha, his3Δ1, leu2Δ0, met15Δ0, ura3Δ0 [brl1Δ::KanMX6] [pRS423-brl1-1::HIS3, 2μ]*	This study
NY3519	DLY1665	*MAT a, his3Δ1, leu2Δ0, met15Δ0, ura3Δ0 [nup100Δ::KanMX4]*	This study
NY3520	SCY4527	*MAT a, his3Δ1, leu2Δ0, met15Δ0, ura3Δ0 [atg1Δ::KanMX4]*	This study
NY3521	SCY4323	*MAT a, ura3-52, leu2-3,112, his3Δ200 [apq12Δ::His3MX6] [scs2Δ::KanMX6] [scs22Δ::His3MX6]*	This study
NY3522	SCY4719	*MAT a, his3Δ1, leu2Δ0, met15Δ0, ura3Δ0 [SEC63-GFP::LEU2]*	This study
NY3523	SCY4720	*MAT a, his3Δ1, leu2Δ0, met15Δ0, ura3Δ0 [atg40Δ::KanMX4] [SEC63-GFP::LEU2]*	This study
NY3524	SCY5093	*MAT a, his3Δ1, leu2Δ0, met15Δ0, ura3Δ0 [apq12Δ::KanMX4] [SEC63-GFP::LEU2]*	This study
NY3525	DLY1698	*MAT a, his3Δ1, leu2Δ0, met15Δ0, ura3Δ0 [brr6-1-3xFLAG::HIS3] [SEC63-GFP::LEU2]*	This study
NY3526	DLY1878	*MAT alpha, his3Δ1, leu2Δ0, met15Δ0, ura3Δ0 [brl1Δ::KanMX6] [pRS423-brl1-1::HIS3, 2μ] [SEC63-GFP::LEU2]*	This study
NY3527	DLY1699	*MAT a, his3Δ1, leu2Δ0, met15Δ0, ura3Δ0 [nup100Δ::KanMX4] [SEC63-GFP::LEU2]*	This study
NY3528	DLY1860	*MAT a, his3Δ1, leu2Δ0, met15Δ0, ura3Δ0 [PER33-GFP::LEU2]*	This study
NY3529	DLY1861	*MAT a, his3Δ1, leu2Δ0, met15Δ0, ura3Δ0 [atg39Δ::KanMX4] [PER33-GFP::LEU2]*	This study
NY3530	DLY1862	*MAT a, his3Δ1, leu2Δ0, met15Δ0, ura3Δ0 [atg40Δ::KanMX4] [PER33-GFP::LEU2]*	This study
NY3531	DLY1863	*MAT a, his3Δ1, leu2Δ0, met15Δ0, ura3Δ0 [apq12Δ::KanMX4] [PER33-GFP::LEU2]*	This study
NY3532	DLY1864	*MAT a, his3Δ1, leu2Δ0, met15Δ0, ura3Δ0 [brr6-1-3xFLAG::HIS3] [PER33-GFP::LEU2]*	This study
NY3533	DLY1865	*MAT alpha, his3Δ1, leu2Δ0, met15Δ0, ura3Δ0 [brl1Δ::KanMX6] [pRS423-brl1-1::HIS3, 2μ] [PER33-GFP::LEU2]*	This study
NY3534	SCY5246	*MAT a, his3Δ1, leu2Δ0, met15Δ0, ura3Δ0 [LNP1-GFP::LEU2]*	This study
NY3535	SCY5247	*MAT a, his3Δ1, leu2Δ0, met15Δ0, ura3Δ0 [atg40Δ::KanMX4] [LNP1-GFP::LEU2]*	This study
NY3536	SCY5248	*MAT a, his3Δ1, leu2Δ0, met15Δ0, ura3Δ0 [apq12Δ::KanMX4] [LNP1-GFP::LEU2]*	This study
NY3537	SCY4603	*MAT a, his3Δ1, leu2Δ0, met15Δ0, ura3Δ0 [RTN1-GFP::LEU2]*	This study
NY3538	SCY4604	*MAT a, his3Δ1, leu2Δ0, met15Δ0, ura3Δ0 [atg40Δ::KanMX4] [RTN1-GFP::LEU2]*	This study
NY3539	SCY5092	*MAT a, his3Δ1, leu2Δ0, met15Δ0, ura3Δ0 [apq12Δ::KanMX4] [RTN1-GFP::LEU2]*	This study
NY3460	NY3460	*MATa, leu2-3, 112, his3Δ200, ura3-52, Gal+ [RTN1-GFP::LEU2]*	Novick lab collection
NY3540	SCY3740	*MAT a, ura3-52, leu2-3,112, his3Δ200 [apq12Δ::KanMX6] [RTN1-GFP::LEU2]*	This study
NY3331	NY3331	*MAT a, leu2-3, 112, his3Δ200, ura3-52, Gal+ [scs2Δ::KanMX6] [scs22Δ::HisMX6] [RTN1-GFP::LEU2]*	Novick lab collection
NY3541	DLY1737	*MAT a, ura3-52, leu2-3,112, his3Δ200 [apq12Δ::His3MX6] [scs2Δ::KanMX6] [scs22Δ::His3MX6] [RTN1-GFP::LEU2]*	This study
SFNY3139	SFNY3139	*MAT a, ura3-52, leu2-3,112, his3Δ200 [atg40Δ::KanMX6] [RTN1-GFP::LEU2]*	Ferro-Novick lab collection
NY3542	SCY4549	*MAT a, his3Δ1, leu2Δ0, met15Δ0, ura3Δ0 [HMG1-GFP::LEU2]*	This study
NY3543	SCY4550	*MAT a, his3Δ1, leu2Δ0, met15Δ0, ura3Δ0 [atg39Δ::KanMX4] [HMG1-GFP::LEU2]*	This study
NY3544	DLY1643	*MAT a, his3Δ1, leu2Δ0, met15Δ0, ura3Δ0 [apq12Δ::KanMX4] [HMG1-GFP::LEU2]*	This study

NY3545	DLY1781	MAT a, his3Δ1, leu2Δ0, met15Δ0, ura3Δ0 [apq12Δ::KanMX4] [apq12-ah-3xHA::HIS3] [HMG1-GFP::LEU2]	This study
NY3546	DLY1694	MAT a, his3Δ1, leu2Δ0, met15Δ0, ura3Δ0 [brr6-1-3xFLAG::HIS3] [HMG1-GFP::LEU2]	This study
NY3547	DLY1820	MAT a, his3Δ1, leu2Δ0, met15Δ0, ura3Δ0 [brl1Δ::KanMX6] [pRS423-brl1-1::HIS3, 2u] [HMG1-GFP::LEU2]	This study
NY3548	DLY1695	MAT a, his3Δ1, leu2Δ0, met15Δ0, ura3Δ0 [nup100Δ::KanMX4] [HMG1-GFP::LEU2]	This study
NY3549	SCY4998	MAT a, his3Δ1, leu2Δ0, met15Δ0, ura3Δ0 [ypt7Δ::His3MX6] [atg8Δ::His3MX6] [GFP-ATG8::LEU2]	This study
NY3550	DLY1684	MAT a, his3Δ1, leu2Δ0, met15Δ0, ura3Δ0 [ypt7Δ::His3MX6] [atg8Δ::His3MX6] [GFP-ATG8::LEU2] [apq12Δ::KanMX6]	This study
SFNY2952	SFNY2952	MAT a, his3Δ1, leuΔ0, met15Δ0, ura3Δ0 [pRS416-GFP-Atg8::URA3, CEN]	Ferro-Novick lab collection
NY3551	DLY1639	MAT a, his3Δ1, leu2Δ0, met15Δ0, ura3Δ0 [atg1Δ::KanMX4] [pRS416-GFP-Atg8::URA3, CEN]	This study
NY3552	DLY1635	MAT a, his3Δ1, leu2Δ0, met15Δ0, ura3Δ0 [apq12Δ::KanMX4] [pRS416-GFP-Atg8::URA3, CEN]	This study
NY3553	DLY1870	MAT a, his3Δ1, leu2Δ0, met15Δ0, ura3Δ0 [brr6-1-3xFLAG::HIS3] [pRS416-GFP-Atg8::URA3, CEN]	This study
NY3554	DLY1869	MAT a, his3Δ1, leu2Δ0, met15Δ0, ura3Δ0 [brl1Δ::KanMX6] [pRS423-brl1-1::HIS3, 2u] [pRS416-GFP-Atg8::URA3, CEN]	This study
NY3555	DLY1674	MAT a, his3Δ1, leu2Δ0, met15Δ0, ura3Δ0 [pho8Δ60::URA3]	This study
NY3556	DLY1675	MAT a, his3Δ1, leu2Δ0, met15Δ0, ura3Δ0 [apq12Δ::KanMX4] [pho8Δ60::URA3]	This study
NY3557	DLY1676	MAT a, his3Δ1, leu2Δ0, met15Δ0, ura3Δ0 [atg1Δ::KanMX4] [pho8Δ60::URA3]	This study
NY3558	SCY5001	MAT a, his3Δ1, leu2Δ0, met15Δ0, ura3Δ0 [OM45-GFP::LEU2]	This study
NY3559	DLY1645	MAT a, his3Δ1, leu2Δ0, met15Δ0, ura3Δ0 [apq12Δ::KanMX4] [OM45-GFP::LEU2]	This study
NY3560	SCY5002	MAT a, his3Δ1, leu2Δ0, met15Δ0, ura3Δ0 [atg32Δ::KanMX4] [OM45-GFP::LEU2]	This study
NY3561	SCY4701	MAT a, his3Δ1, leu2Δ0, met15Δ0, ura3Δ0 [ATG40-GFP::LEU2]	This study
NY3562	SCY5116	MAT a, his3Δ1, leu2Δ0, met15Δ0, ura3Δ0 [atg11Δ::KanMX4] [ATG40-GFP::LEU2]	This study
NY3563	SCY5119	MAT a, his3Δ1, leu2Δ0, met15Δ0, ura3Δ0 [apq12Δ::KanMX4] [ATG40-GFP::LEU2]	This study
NY3564	DLY1679	MAT a, his3Δ1, leu2Δ0, met15Δ0, ura3Δ0 [ATG39-GFP-ATG39 3'UTR::LEU2]	This study
NY3565	DLY1680	MAT a, his3Δ1, leu2Δ0, met15Δ0, ura3Δ0 [apq12Δ::KanMX4] [ATG39-GFP-ATG39 3'UTR::LEU2]	This study
NY3566	DLY1688	MAT a, his3Δ1, leu2Δ0, met15Δ0, ura3Δ0 [SRC1-GFP::LEU2]	This study
NY3567	DLY1689	MAT a, his3Δ1, leu2Δ0, met15Δ0, ura3Δ0 [atg39Δ::KanMX4] [SRC1-GFP::LEU2]	This study
NY3568	DLY1690	MAT a, his3Δ1, leu2Δ0, met15Δ0, ura3Δ0 [apq12Δ::KanMX4] [SRC1-GFP::LEU2]	This study
NY3569	DLY1783	MAT a, his3Δ1, leu2Δ0, met15Δ0, ura3Δ0 [apq12Δ::KanMX4] [apq12-ah-3xHA::HIS3] [SRC1-GFP::LEU2]	This study
NY3570	DLY1691	MAT a, his3Δ1, leu2Δ0, met15Δ0, ura3Δ0 [brr6-1-3xFLAG::HIS3] [SRC1-GFP::LEU2]	This study
NY3571	DLY1822	MAT alpha, his3Δ1, leu2Δ0, met15Δ0, ura3Δ0 [brl1Δ::KanMX6] [pRS423-brl1-1::HIS3, 2μ] [SRC1-GFP::LEU2]	This study
NY3572	DLY1692	MAT a, his3Δ1, leu2Δ0, met15Δ0, ura3Δ0 [nup100Δ::KanMX4] [SRC1-GFP::LEU2]	This study
NY3573	DLY1753	MAT a, his3Δ1, leu2Δ0, met15Δ0, ura3Δ0 [NUP85-GFP::LEU2]	This study
NY3574	DLY1754	MAT alpha, his3Δ1, leu2Δ0, met15Δ0, ura3Δ0 [atg1Δ::NatMX6] [NUP85-GFP::LEU2]	This study
NY3575	DLY1755	MAT a, his3Δ1, leu2Δ0, met15Δ0, ura3Δ0 [apq12Δ::KanMX4] [NUP85-GFP::LEU2]	This study
NY3576	DLY1757	MAT a, his3Δ1, leu2Δ0, met15Δ0, ura3Δ0 [NUP159-GFP::LEU2]	This study

NY3577	DLY1758	*MAT alpha, his3Δ1, leu2Δ0, met15Δ0, ura3Δ0, [atg1Δ::NatMX6] [NUP159-GFP::LEU2]*	This study
NY3578	DLY1759	*MAT a, his3Δ1, leu2Δ0, met15Δ0, ura3Δ0, [apq12Δ::KanMX4] [NUP159-GFP::LEU2]*	This study
NY3579	DLY1670	*MAT a his3Δ1, leu2Δ0, met15Δ0, ura3Δ0 [pRS315-NVJ1-GFP::LEU2, CEN]*	This study
NY3580	DLY1671	*MAT a, his3Δ1, leu2Δ0, met15Δ0, ura3Δ0 [atg39Δ::KanMX4] [pRS315-NVJ1-GFP::LEU2, CEN]*	This study
NY3581	DLY1672	*MAT a, his3Δ1, leu2Δ0, met15Δ0, ura3Δ0 [apq12Δ::KanMX4] [pRS315-NVJ1-GFP::LEU2, CEN]*	This study
NY3582	DLY1824	*MAT a, his3Δ1, leu2Δ0, met15Δ0, ura3Δ0 [APQ12-GFP::LEU2]*	This study
NY3583	DLY1825	*MAT a, his3Δ1, leu2Δ0, met15Δ0, ura3Δ0 [atg39Δ::KanMX4] [APQ12-GFP::LEU2]*	This study
NY3584	DLY1859	*MAT a, his3Δ1, leu2Δ0, met15Δ0, ura3Δ0 [atg40Δ::KanMX4] [APQ12-GFP::LEU2]*	This study
NY3585	DLY1826	*MAT a, his3Δ1, leu2Δ0, met15Δ0, ura3Δ0 [BRL1-GFP::LEU2]*	This study
NY3586	DLY1827	*MAT a, his3Δ1, leu2Δ0, met15Δ0, ura3Δ0 [atg39Δ::KanMX4] [BRL1-GFP::LEU2]*	This study
NY3587	DLY1742	*MAT a, his3Δ1, leu2Δ0, met15Δ0, ura3Δ0 [apq12Δ::KanMX4] [APQ12-3xHA::LEU2]*	This study
NY3588	DLY1740	*MAT a, his3Δ1, leu2Δ0, met15Δ0, ura3Δ0 [apq12Δ::KanMX4] [APQ12-3xHA::LEU2] [ATG39-3xFLAG::URA3]*	This study
NY3589	DLY1741	*MAT a, his3Δ1, leu2Δ0, met15Δ0, ura3Δ0 [apq12Δ::KanMX4] [APQ12-3xHA::LEU2] [pCUP1-ATG39-3xFLAG::URA3, 2μ]*	This study
NY3590	DLY1763	*MAT a, his3Δ1, leu2Δ0, met15Δ0, ura3Δ0 [BRL1-3xHA::LEU2]*	This study
NY3591	DLY1771	*MAT a, his3Δ1, leu2Δ0, met15Δ0, ura3Δ0 [BRL1-3xHA::LEU2] [ATG39-3xFLAG-ATG39 3'UTR::URA3]*	This study
NY3592	DLY1772	*MAT a, his3Δ1, leu2Δ0, met15Δ0, ura3Δ0 [BRL1-3xHA::LEU2] [pCUP1-ATG39-3xFLAG::URA3, 2μ]*	This study
NY3593	DLY1773	*MAT a, his3Δ1, leu2Δ0, met15Δ0, ura3Δ0 [BRR6-3xHA::LEU2]*	This study
NY3594	DLY1774	*MAT a, his3Δ1, leu2Δ0, met15Δ0, ura3Δ0 [ATG39-3xFLAG-ATG39 3'UTR::URA3] [BRR6-3xHA::LEU2]*	This study
NY3595	DLY1775	*MAT a, his3Δ1, leu2Δ0, met15Δ0, ura3Δ0 [pCUP1-ATG39-3xFLAG::URA3, 2μ] [BRR6-3xHA::LEU2]*	This study
NY3596	DLY1677	*MAT a, his3Δ1, leu2Δ0, met15Δ0, ura3Δ0 [apq12Δ::KanMX4] [APQ12-3xEGFP::LEU2]*	This study
NY3597	SCY5276	*MAT a, his3Δ1, leu2Δ0, met15Δ0, ura3Δ0 [LNP1-3xEGFP::LEU2] [RTN1-mCherry::URA3]*	This study
NY3598	SCY5277	*MAT a, his3Δ1, leu2Δ0, met15Δ0, ura3Δ0 [apq12Δ::KanMX4] [LNP1-3xEGFP::LEU2] [RTN1-mCherry::URA3]*	This study
NY3599	HYY1035	*MAT a, his3Δ1, leu2Δ0, met15Δ0, ura3Δ0 [SRC1-GFP::LEU2] [pRS316-pRtn1-ATG39-3xmCherry::URA3, CEN]*	This study
NY3600	HYY1036	*MAT a, his3Δ1, leu2Δ0, met15Δ0, ura3Δ0 [apq12Δ::KanMX4] [SRC1-GFP::LEU2] [pRS316-pRtn1-ATG39-3xmCherry::URA3, CEN]*	This study
NY3601	HYY1025	*MAT a, his3Δ1, leu2Δ0, met15Δ0, ura3Δ0 [ATG40-2xmCherry::URA3] [pRS315-GFP-ATG8::LEU2, CEN]*	This study
NY3602	HYY1026	*MAT a, his3Δ1, leu2Δ0, met15Δ0, ura3Δ0 [apq12Δ::KanMX4] [ATG40-2xmCherry::URA3] [pRS315-GFP-ATG8::LEU2, CEN]*	This study
NY3603	HYY1039	*MAT a, his3Δ1, leu2Δ0, met15Δ0, ura3Δ0 [pRS316-pRtn1-ATG39-3xmCherry::URA3, CEN] [pRS315-GFP-ATG8::LEU2, CEN]*	This study
NY3604	HYY1040	*MAT a, his3Δ1, leu2Δ0, met15Δ0, ura3Δ0 [apq12Δ::KanMX4] [pRS316-pRtn1-ATG39-3xmCherry::URA3, CEN] [pRS315-GFP-ATG8::LEU2, CEN]*	This study
NY3605	HYY1020	*MAT a, his3Δ1, leu2Δ0, met15Δ0, ura3Δ0 [apq12Δ::KanMX4] [APQ12-3xEGFP::LEU2] [ATG40-2xmCherry::URA3]*	This study
NY3606	HYY1031	*MAT a, his3Δ1, leu2Δ0, met15Δ0, ura3Δ0 [apq12Δ::KanMX4] [APQ12-3xEGFP::LEU2] [pRS316-pRtn1-ATG39-3xmCherry::URA3, CEN]*	This study

NY3607	DLY1807	*MAT a, his3Δ1, leu2Δ0, met15Δ0, ura3Δ0, [apq12Δ::KanMX4] [APQ12-3xHA::LEU2] [pRS426-pCUP1-Atg40-3xFLAG::URA3, 2u]*	This study
NY3608	DLY1883	*MAT a, his3Δ1, leu2Δ0, met15Δ0, ura3Δ0 [BRL1-3xHA::LEU2] [pRS426-pCUP1-Atg40-3xFLAG::URA3, 2u]*	This study
NY3609	DLY1885	*MAT a, his3Δ1, leu2Δ0, met15Δ0, ura3Δ0 [BRR6-3xHA::LEU2] [pRS426-pCUP1-Atg40-3xFLAG::URA3, 2u]*	This study

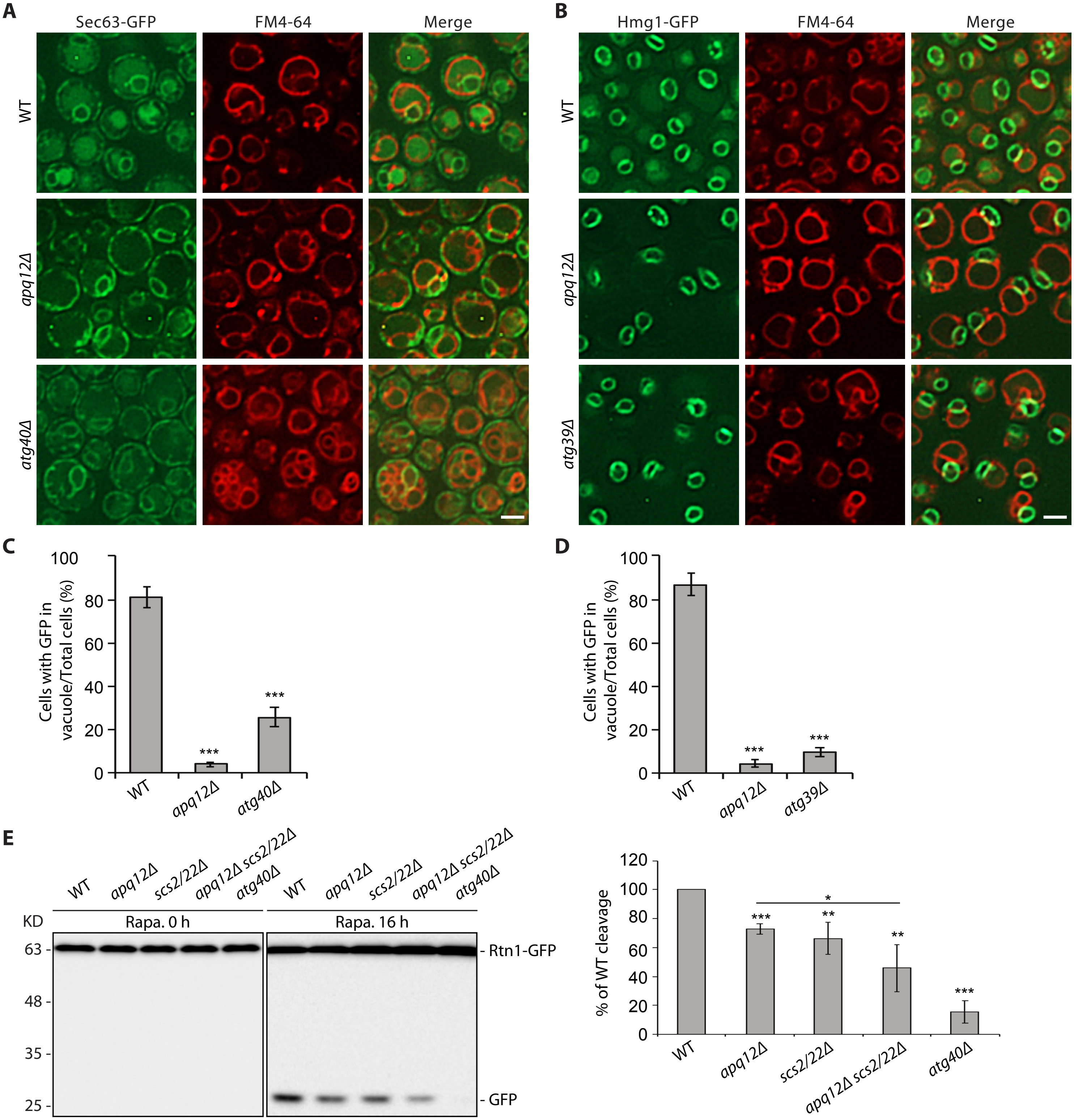

A
Sec63-GFP
FM4-64
Merge
WT
apq12Δ
atg40Δ
B
Hmg1-GFP
FM4-64
Merge
WT
apq12Δ
atg39Δ
C
Cells with GFP in vacuole/Total cells (%)
100
80
60
40
20
0
***
***
WT
apq12Δ
atg40Δ
D
Cells with GFP in vacuole/Total cells (%)
100
80
60
40
20
0
***
***
WT
apq12Δ
atg39Δ
E
WT
apq12Δ
scs2/22Δ
apq12Δ scs2/22Δ
atg40Δ
WT
apq12Δ
scs2/22Δ
apq12Δ scs2/22Δ
atg40Δ
KD
Rapa. 0 h
Rapa. 16 h
63 -
48 -
35 -
25 -
- Rtn1-GFP
- GFP
% of WT cleavage
120
100
80
60
40
20
0
*
***
**
**
***
WT
apq12Δ
scs2/22Δ
apq12Δ scs2/22Δ
atg40Δ

Figure S1

Fig. S1. Apq12 is required for both ER-phagy and nucleophagy. (A) Yeast cells (WT, *apq12Δ*, and *atg40Δ*) expressing Sec63-GFP were treated with rapamycin for 16 h and then imaged after FM4-64 staining of vacuole membranes. Scale bar, 2 μm in all panels. (B) Yeast cells (WT, *apq12Δ*, and *atg39Δ*) expressing Hmg1-GFP were treated with rapamycin for 16 h and then imaged after FM4-64 staining of vacuole membranes. Scale bar, 2 μm in all panels. (C) The percentage of Sec63-GFP–expressing cells showing GFP in the vacuole was quantified. P values were calculated by comparing each mutant with WT using Student's t-test: Error bars represent SD, n = 3 independent experiments. ***, $P < 0.001$. (D) The percentage of Hmg1-GFP–expressing cells showing GFP signal in the vacuole was quantified and statistically analyzed as in (C). (E) Yeast cells expressing Rtn1-GFP were harvested before and after 16 h of rapamycin treatment. Lysates were analyzed by Western blotting using an anti-GFP antibody.

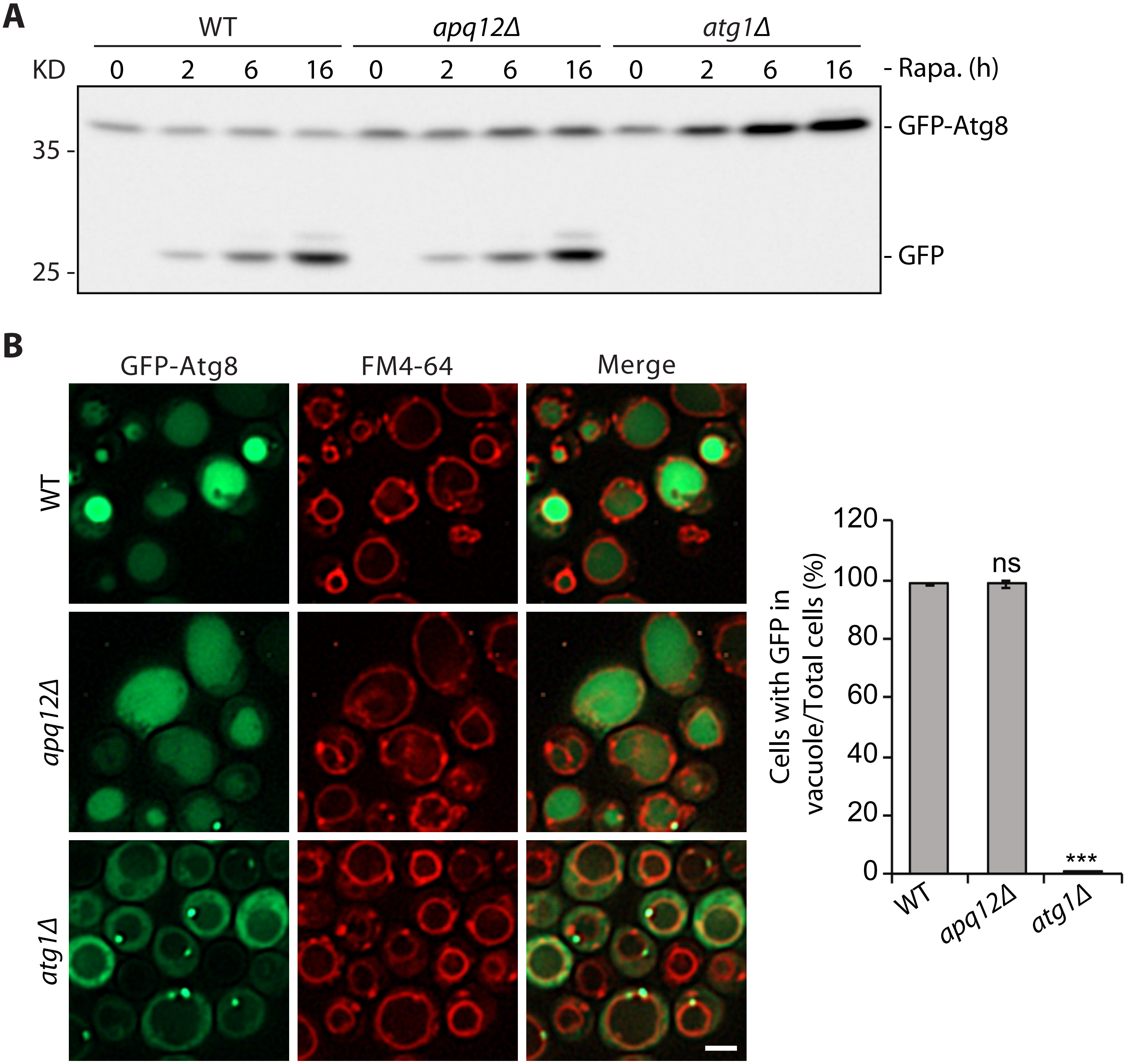
A
WT
apq12Δ
atg1Δ
KD
0
2
6
16
0
2
6
16
0
2
6
16
- Rapa. (h)
35 -
25 -
- GFP-Atg8
- GFP
B
GFP-Atg8
FM4-64
Merge
WT
apq12Δ
atg1Δ
Cells with GFP in vacuole/Total cells (%)
120
100
80
60
40
20
0
ns
***
WT
apq12Δ
atg1Δ

Figure S2

Fig. S2. Apq12 is dispensable for bulk autophagy. (A) Yeast cells (WT, *apq12Δ*, and *atg1Δ*) expressing GFP-Atg8 were harvested before (0 h) and after rapamycin treatment (for 2, 6, and 16 h), followed by Western blot analysis using an anti-GFP antibody. (B) Yeast cells expressing GFP-Atg8 were treated with rapamycin for 16 h, vacuole membranes were stained with FM4-64 and imaged. Scale bar, 2 μm in all panels. The percentage of cells showing GFP signal in the vacuole was quantified. P values were calculated by comparing each mutant with WT using Student's t-test: Error bars represent SD, n = 3 independent experiments. ns, not significant ($P \geq 0.05$); ***, $P < 0.001$.

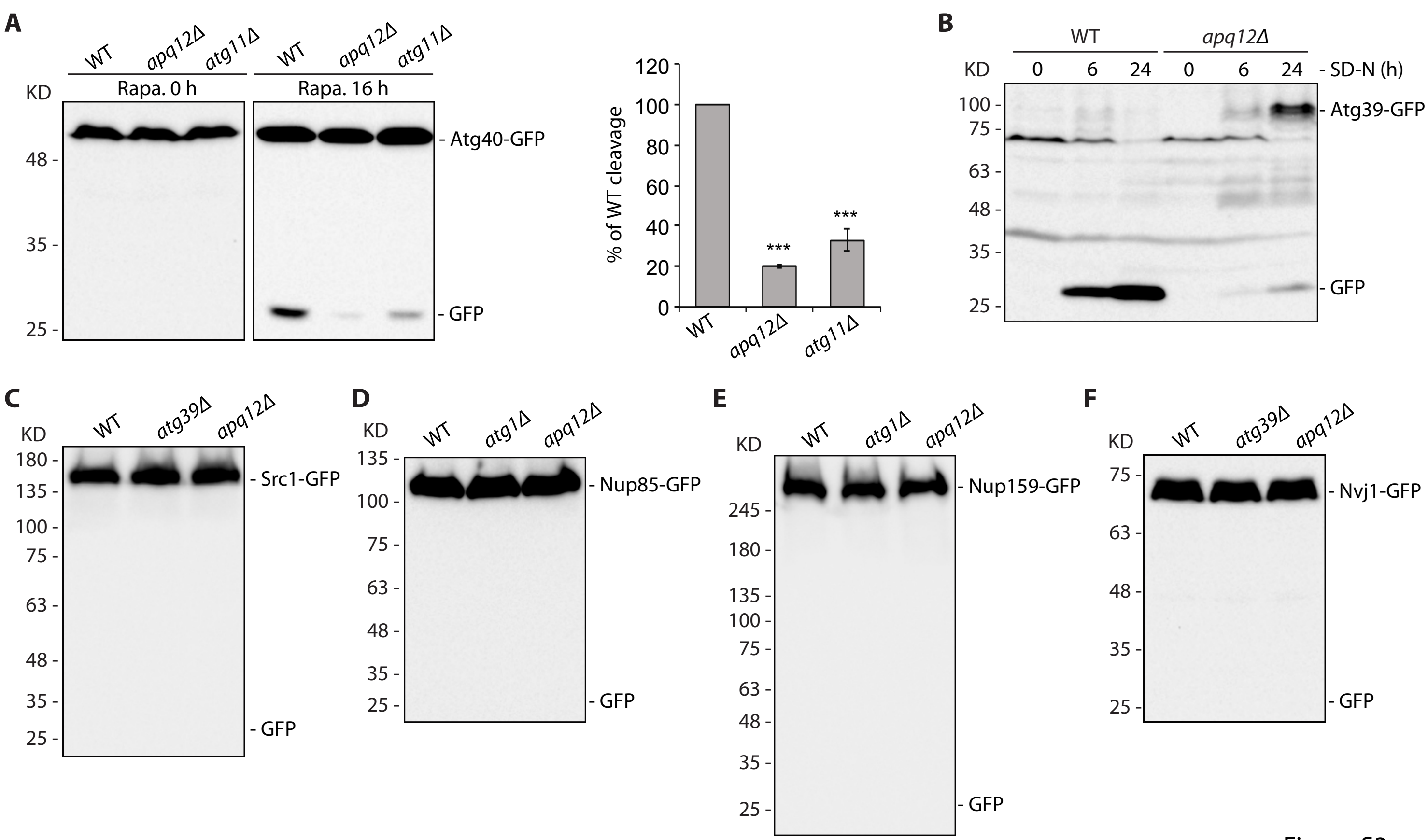

A
WT
apq12Δ
atg11Δ
Rapa. 0 h
Rapa. 16 h
KD
48
35
25
Atg40-GFP
GFP
% of WT cleavage
120
100
80
60
40
20
0
***
B
apq12Δ
SD-N (h)
0
6
24
Atg39-GFP
C
atg39Δ
Src1-GFP
D
atg1Δ
Nup85-GFP
E
Nup159-GFP
F
Nvj1-GFP

Figure S3

Fig. S3. Apq12 is required for autophagic degradation of Atg40, Atg39 and its cargo. (A) Yeast cells expressing Atg40-GFP were harvested before and after 16 h of rapamycin treatment, and GFP cleavage was analyzed by Western blotting using anti-GFP antibodies (left panels). The percentage of free GFP relative to total GFP was quantified for the 16 h rapamycin-treated samples (right panels), with WT set to 100% and all mutants normalized to the WT control. Error bars represent SD (N = 3 independent experiments). P values were calculated by comparing each mutant with WT using Student's t-test: ***, $P < 0.001$. (B) GFP cleavage was analyzed in cells expressing Atg39-GFP after 0, 6 or 24 h of nitrogen starvation. Cell lysates were examined by Western blotting using an anti-GFP antibody. (C-F) GFP cleavage was analyzed in cells expressing different GFP-tagged proteins before rapamycin treatment (C, Src1-GFP; D, Nup85-GFP; E, Nup159-GFP; F, Nvj1-GFP). Cell lysates were examined by Western blotting using an anti-GFP antibody.

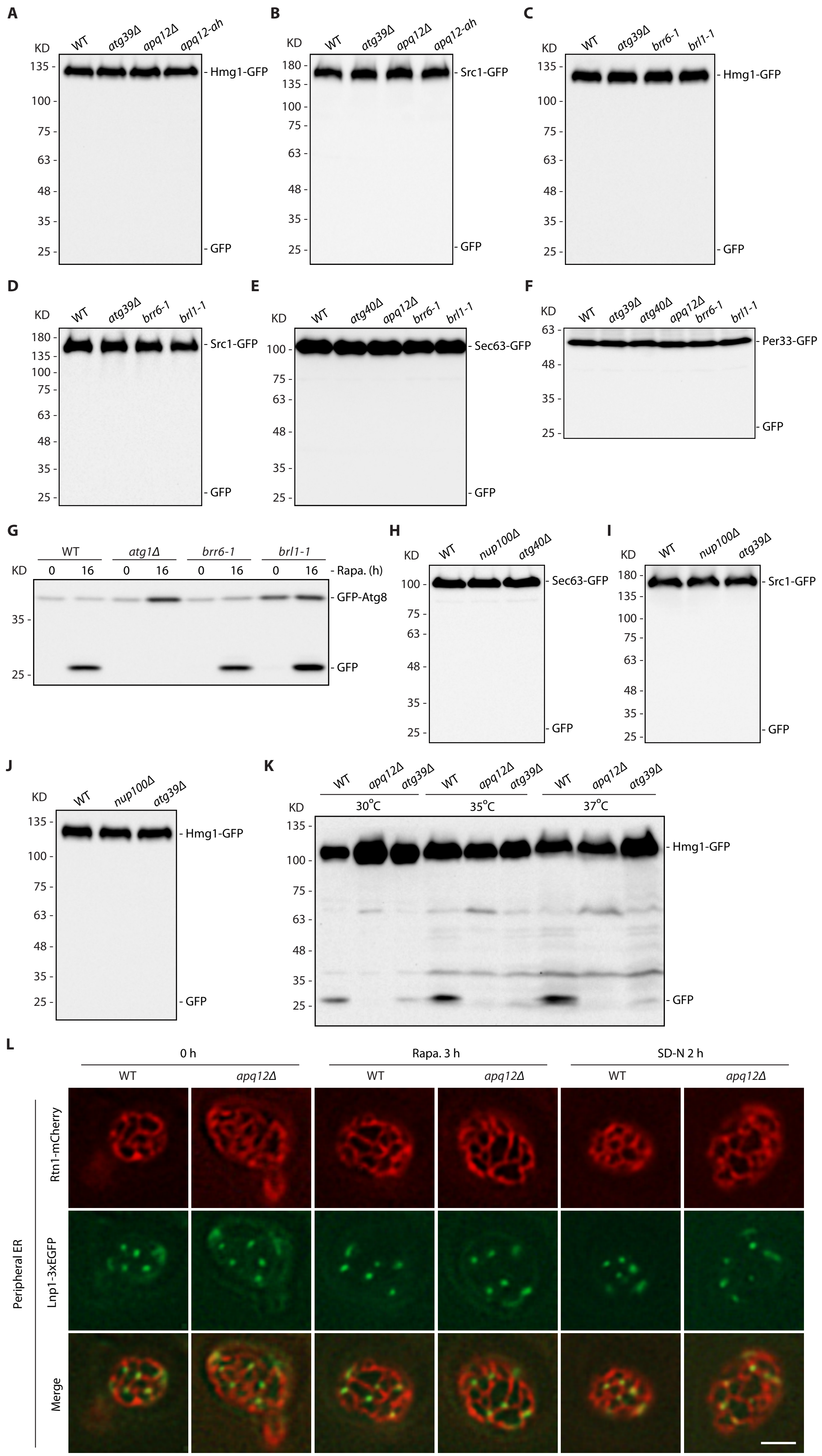

A
KD
WT
atg39Δ
apq12Δ
apq12-ah
135
100
75
63
48
35
25
Hmg1-GFP
GFP
B
KD
WT
atg39Δ
apq12Δ
apq12-ah
180
135
100
75
63
48
35
25
Src1-GFP
GFP
C
KD
WT
atg39Δ
brr6-1
brl1-1
135
100
75
63
48
35
25
Hmg1-GFP
GFP
D
KD
WT
atg39Δ
brr6-1
brl1-1
180
135
100
75
63
48
35
25
Src1-GFP
GFP
E
KD
WT
atg40Δ
apq12Δ
brr6-1
brl1-1
100
75
63
48
35
25
Sec63-GFP
GFP
F
KD
WT
atg39Δ
atg40Δ
apq12Δ
brr6-1
brl1-1
63
48
35
25
Per33-GFP
GFP
G
WT
atg1Δ
brr6-1
brl1-1
KD
0
16
Rapa. (h)
35
25
GFP-Atg8
GFP
H
KD
WT
nup100Δ
atg40Δ
100
75
63
48
35
25
Sec63-GFP
GFP
I
KD
WT
nup100Δ
atg39Δ
180
135
100
75
63
48
35
25
Src1-GFP
GFP
J
KD
WT
nup100Δ
atg39Δ
135
100
75
63
48
35
25
Hmg1-GFP
GFP
K
WT
apq12Δ
atg39Δ
30°C
35°C
37°C
KD
135
100
75
63
48
35
25
Hmg1-GFP
GFP
L
0 h
Rapa. 3 h
SD-N 2 h
WT
apq12Δ
Peripheral ER
Rtn1-mCherry
Lnp1-3xEGFP
Merge

Figure S4

Fig. S4. Apq12 works with Brl1 and Brr6 on a pathway independent of nuclear pore assembly. (A–K) GFP cleavage was analyzed by Western blot using an anti-GFP antibody of cells expressing the indicated GFP-tagged proteins before or after 16 h of rapamycin treatment. Panels A–J show cells before rapamycin treatment (A, Hmg1-GFP; B, Src1-GFP; C, Hmg1-GFP; D, Src1-GFP; E, Sec63-GFP; F, Per33-GFP; G, GFP-Atg8 (0 h); H, Sec63-GFP; I, Src1-GFP; J, Hmg1-GFP), whereas panels G and K show cells after 16 h of rapamycin treatment (G, GFP-Atg8; K, Hmg1-GFP). (K) Yeast cells expressing Hmg1-GFP were cultured at 30°C, 35°C, or 37°C. Unless otherwise indicated, cells were cultured at 30°C. (L) Peripheral ER structure is normal in *apq12Δ* cells. WT and *apq12Δ* yeast cells co-expressing Rtn1-mCherry and Lnp1-3×EGFP were left untreated or subjected to rapamycin treatment (3 h) or nitrogen starvation in SD-N medium (2 h) before imaging. Scale bar, 2 μm in all panels.

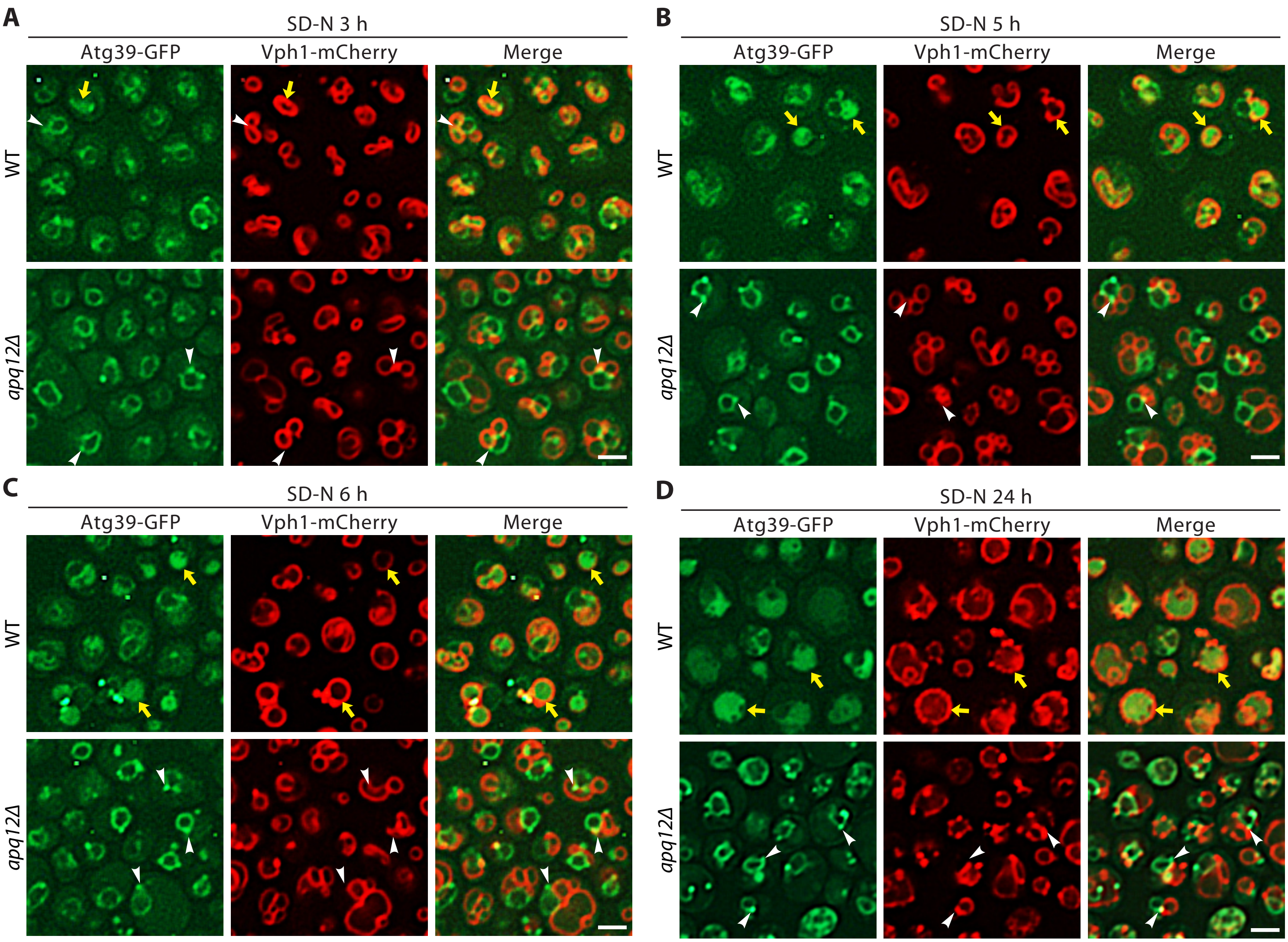
A
SD-N 3 h
Atg39-GFP
Vph1-mCherry
Merge
WT
apq12Δ
B
SD-N 5 h
Atg39-GFP
Vph1-mCherry
Merge
WT
apq12Δ
C
SD-N 6 h
Atg39-GFP
Vph1-mCherry
Merge
WT
apq12Δ
D
SD-N 24 h
Atg39-GFP
Vph1-mCherry
Merge
WT
apq12Δ

Figure S5

Fig. S5. Atg39–GFP extensions accumulate in *apq12Δ* cells. Fluorescence images of WT and *apq12Δ* cells co-expressing Atg39-GFP and Vph1-mCherry after nitrogen starvation for 3, 5, 6, and 24 h. Arrowheads indicate Atg39-GFP extensions from the nuclear membrane into the cytoplasm or bright puncta on the nuclear membrane, whereas arrows indicate Atg39-GFP signals within the vacuole. Scale bar, 2 μm in all panels. Panels: (A) SD–N, 3 h; (B) 5 h; (C) 6 h; (D) 24 h.

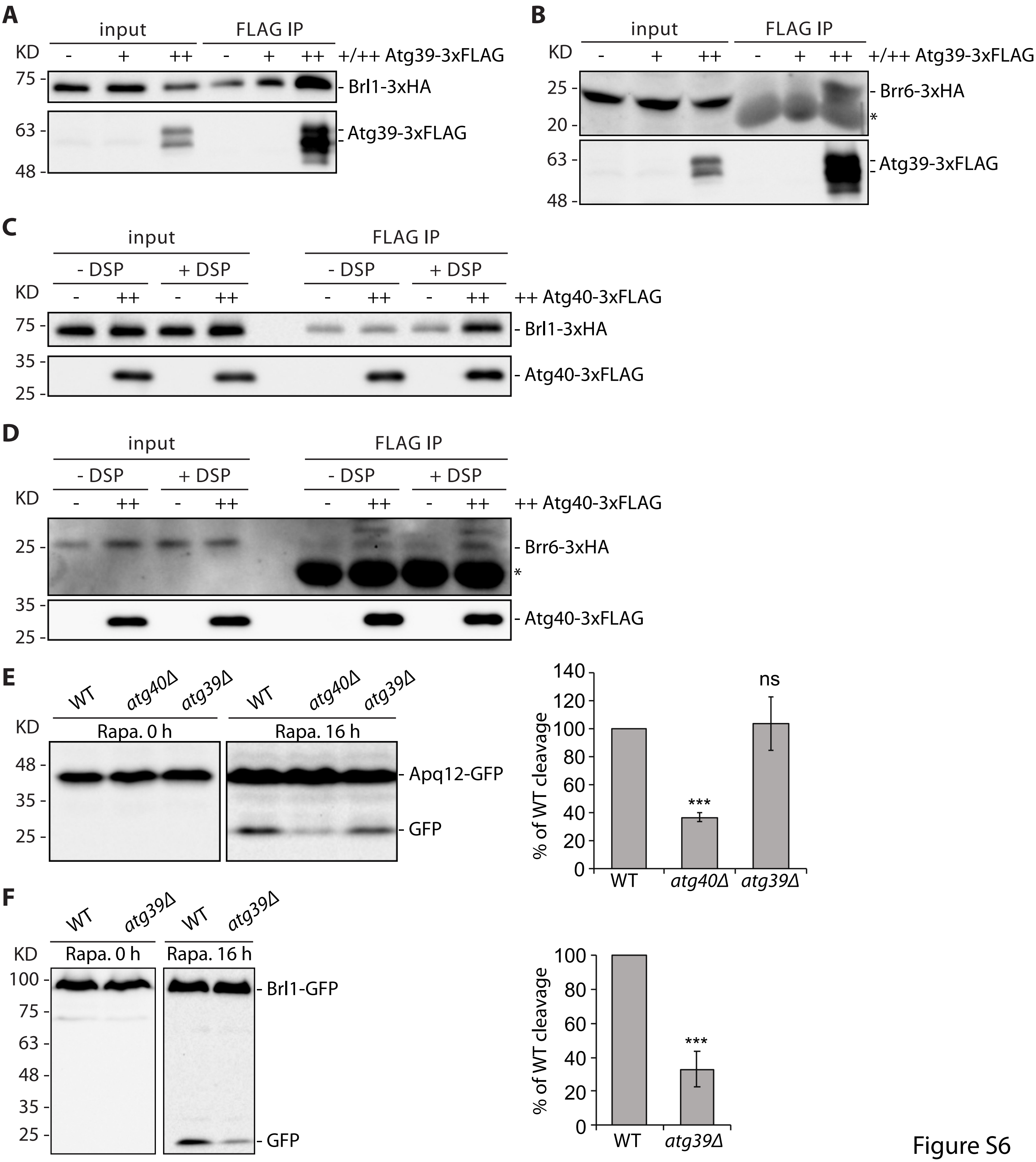
A
input
FLAG IP
KD
- + ++ - + ++
+/++ Atg39-3xFLAG
75
63
48
Brl1-3xHA
Atg39-3xFLAG
B
input
FLAG IP
KD
- + ++ - + ++
+/++ Atg39-3xFLAG
25
20
63
48
Brr6-3xHA
*
Atg39-3xFLAG
C
input
FLAG IP
- DSP
+ DSP
- DSP
+ DSP
KD
- ++ - ++ - ++ - ++
++ Atg40-3xFLAG
75
35
25
Brl1-3xHA
Atg40-3xFLAG
D
input
FLAG IP
- DSP
+ DSP
- DSP
+ DSP
KD
- ++ - ++ - ++ - ++
++ Atg40-3xFLAG
25
35
25
Brr6-3xHA
*
Atg40-3xFLAG
E
WT
atg40Δ
atg39Δ
WT
atg40Δ
atg39Δ
KD
Rapa. 0 h
Rapa. 16 h
48
35
25
Apq12-GFP
GFP
% of WT cleavage
140
120
100
80
60
40
20
0
ns
***
WT
atg40Δ
atg39Δ
F
WT
atg39Δ
WT
atg39Δ
KD
Rapa. 0 h
Rapa. 16 h
100
75
63
48
35
25
Brl1-GFP
GFP
% of WT cleavage
100
80
60
40
20
0
***
WT
atg39Δ

Figure S6

Fig. S6. Brl1 and Brr6 associate with Atg39 and Atg40. (A-B) WT cells expressing (A) Brl1-3xHA or (B) Brr6-3xHA together with Atg39-3xFLAG at none (−), endogenous (+), or overexpressed level under the copper promoter (++), as indicated in the figure, were incubated with rapamycin for 3 h and then treated with the crosslinking reagent DSP. Cells were lysed and immunoprecipitated using anti-FLAG beads (see Materials and Methods). Input samples and precipitates were analyzed by Western blotting using an anti-HA antibody to detect Brl1-3xHA or Brr6-3xHA and an anti-FLAG antibody to detect Atg39-3xFLAG. The input was 0.1% for Brl1-3xHA, 0.5% for Brr6-3xHA, and 10% for Atg39-3xFLAG. For overexpression of Atg39-3xFLAG under the copper promoter, no additional $CuSO_4$ was added to the medium. * indicates a non-specific band from IgG light chain. (C-D) WT cells expressing (C) Brl1-3xHA or (D) Brr6-3xHA together with Atg40-3xFLAG at none (-) or overexpressed level under the *CUP1* promoter (++; 125 μM $CuSO_4$), as indicated in the figure, were incubated with rapamycin for 3 h and then treated without (− DSP) or with (+ DSP) the crosslinking reagent DSP. Cells were lysed and immunoprecipitated as described in (A). The input was 0.2% for Brl1-3xHA, 0.25% for Brr6-3xHA, and 50% for Atg40-3xFLAG. (E-F) Yeast cells expressing Apq12-GFP (E) or Brl1-GFP (F) were harvested before and after 16 h of rapamycin treatment, and GFP cleavage was analyzed by Western blotting using anti-GFP antibodies (left panels). The percentage of free GFP relative to total GFP was quantified for the 16 h rapamycin-treated samples (right panels), with WT set to 100% and all mutants normalized to the WT control. Error bars represent SD (N = 3 independent experiments). P values were calculated by comparing each mutant with WT using Student's t-test: ns, not significant ($P \geq 0.05$); ***, $P < 0.001$.
